# The NnaR orphan response regulator is essential for the utilization of nitrate and nitrite as sole nitrogen sources in mycobacteria

**DOI:** 10.1038/s41598-018-35844-z

**Published:** 2018-12-03

**Authors:** Magdalena Antczak, Renata Płocińska, Przemysław Płociński, Anna Rumijowska-Galewicz, Anna Żaczek, Dominik Strapagiel, Jarosław Dziadek

**Affiliations:** 1 Institute for Medical Biology, Polish Academy of Sciences, Łódź, Poland; 2 Department of Biochemistry and Cell Biology, University of Rzeszów, Rzeszów, Poland; 3 Biobank Lab, Department of Molecular Biophysics, University of Łódź, Łódź, Poland

## Abstract

Nitrogen is an essential component of biological molecules and an indispensable microelement required for the growth of cells. Nitrogen metabolism of *Mycobacterium smegmatis* is regulated by a number of transcription factors, with the *glnR* gene product playing a major role. Under nitrogen-depletion conditions, GlnR controls the expression of many genes involved in nitrogen assimilation, including the *msmeg_0432* gene encoding NnaR, the homologue of a nitrite/nitrate transport regulator from *Streptomyces coelicolor*. In the present study, the role of NnaR in the nitrogen metabolism of *M*. *smegmatis* was evaluated. The ∆*glnR* and ∆*nnaR* mutant strains were generated and cultured under nitrogen-depletion conditions. Total RNA profiling was used to investigate the potential role of NnaR in the GlnR regulon under nitrogen-depletion and in nitrogen-rich media. We found that disruption of MSMEG_0432 affected the expression of genes involved in nitrite/nitrate uptake, and its removal rendered mycobacteria unable to assimilate nitrogen from those sources, leading to cell death. RNA-Seq results were validated using quantitative real-time polymerase chain reaction (qRT-PCR) and electrophoretic mobility shift assays (EMSAs). The ability of mutants to grow on various nitrogen sources was evaluated using the BIOLOG Phenotype screening platform and confirmed on minimal Sauton’s medium containing various sources of nitrogen. The ∆*glnR* mutant was not able to convert nitrates to nitrites. Interestingly, NnaR required active GlnR to prevent nitrogen starvation, and both proteins cooperated in the regulation of gene expression associated with nitrate/nitrite assimilation. The ∆*nnaR* mutant was able to convert nitrates to nitrites, but it could not assimilate the products of this conversion. Importantly, NnaR was the key regulator of the expression of the truncated haemoglobin trHbN, which is required to improve the survival of bacteria under nitrosative stress.

## Introduction

The genus *Mycobacterium* contains obligatory pathogens known to cause serious diseases in mammals, including tuberculosis (*Mycobacterium tuberculosis*, *Mtb*) and leprosy (*M*. *leprae*) in humans, as well as a large number of opportunistic pathogens and/or free-living saprophytes, such as *Mycobacterium smegmatis* (*M*. *smegmatis*)^1^. The growth of bacteria occupying various environmental niches depends on the growth conditions and availability of essential elements such as carbon, oxygen and nitrogen that are used for biosynthesis of proteins, nucleic acids and cell wall components^2^. The genome of *M*. *smegmatis* contains a number of genes believed to be involved in nitrogen metabolism, but the real functions of the majority of them are still unknown. For bacteria, an important source of nitrogen is ammonium entering cells by diffusion across the cytoplasmic membrane or via protein-dependent transport. Three ammonium transporters (Amt1, AmtA, AmtB) have been identified in the cell wall of *M*. *smegmatis*. However, the bacterium also possess genes allowing assimilation of the nitrogen from urea or nitrite. The accessibility of the nitrogen determines the mode of its utilization. Glutamate is the main nitrogen-storage molecule in bacteria. In nitrogen-rich environments, glutamate dehydrogenase (GDH) plays a main role in nitrogen accumulation, as it is able to convert ammonium to L-glutamate. Under nitrogen deficiency, the glutamine synthetase/glutamate synthase (GS/GOGAT) is activated^2–4^. Switching between the pathways is accompanied by changes in gene expression of the *amtB* (*msmeg_2425*) operon, consisting of ammonium transporter AmtB, nitrogen regulatory protein P-II (*msmeg_2426*), and GlnD adenylyl transferase (*msmeg_2427*), as well as two additional ammonium transporters, AmtA (*msmeg_4635*) and Amt1 (*msmeg_6259*), glutamine synthetase and glutamate synthase, encoded by *glnA1* and *gltBD*, respectively. Under nitrogen depletion, GlnD adenylates the GlnK (PII) protein, allowing its dissociation from the AmtB porin channel, resulting in ammonium inflow, and the glutamine synthetase is de-adenylated by GlnE^3,5^.

Two major transcriptional regulators of nitrogen metabolism have been identified in Actinobacteria, AmtR and GlnR. The AmtR of *Corynebacterium glutamicum* regulates more than 30 genes^6^, whereas the GlnR of *Streptomyces coelicolor* (*S*. *coelicolor*) affects the expression of more than 50 genes^4,7–9^. The homologs of both regulators, AmtR (*msmeg_4300*) and GlnR (*msmeg_5784*), were identified in *M*. *smegmatis*, but *Mtb* only encodes GlnR^2^. In both *Mtb* and *M*. *smegmatis*, GlnR is considered a key transcriptional regulator of nitrogen metabolism. GlnR controls the expression of ammonium transporters (*amt1*, *amtB*), signal transduction components (*glnK*, *glnD*), glutamine synthetase (*glnA*) in *M*. *smegmatis*^10^ and *nirBD* expression in *M*. *tuberculosis*^11^. Response of *Mtb* to nitrogen starvation is fairly well characterized, including identification of GlnR’s DNA binding consensus and regulon^5^. Very recently Liu and colleagues demonstrated GlnR mediated regulation of short chain fatty acid synthesis in *M*. *smegmatis*. GlnR was found to bind to promoter regions of *prpE* (AMP- forming propionyl-CoA synthetase) and 4 AMP- forming *acs* genes (acetyl-CoA) under nitrogen starvation^12^. A separate study revealed GlnR’s involvement in the regulation of methylcitrate cycle by directly controlling the expression of *prpDBC* operon^13^. Furthermore, the GlnR-dependent regulation of more than 100 genes under nitrogen depletion has been demonstrated using ChIP-seq technology in *M*. *smegmatis*^3^. Among them are those involved in ammonium, nitrate/nitrite, amino acid/peptide and urea uptake, genes encoding the nitrite reductase NirBD, amine oxidase, urea amidolyase, deaminase and hydrolases acting on carbon-nitrogen bonds, as well as regulatory genes, including *msmeg_0432*, the homologue of the nitrite/nitrate transport regulator (NnaR) of *S*. *coelicolor*^14^ also present in *M*. *tuberculosis*^5^. GlnR and NnaR both belong to the OmpR-type family of two-component signal transduction system elements, acting as orphan response regulators (RRs)^15^. Both GlnR^16^ and NnaR contain the conserved aspartic acid residue that could potentially be phosphorylated by the cognate histidine kinase, but no kinase has been identified as a partner for those regulators. On the other hand, GlnR is phosphorylated by the serine/threonine kinases and is acetylated in *Streptomyces*, and those modifications supposedly regulate the protein’s activity as a response regulator^12,17^. Here, we have engineered Δ*nnaR* and Δ*glnR M*. *smegmatis* mutants to evaluate systematically the role of NnaR as a regulator of nitrogen metabolism in *M*. *smegmatis* by using phenotypic microarray technology and RNA-Seq analysis and by monitoring the kinetics of growth and viability in the presence of various nitrogen sources.

## Results

### The NnaR regulator is not essential for growth or survival of *M*. *smegmatis* cells in nitrogen-rich media

**Figure 1:**
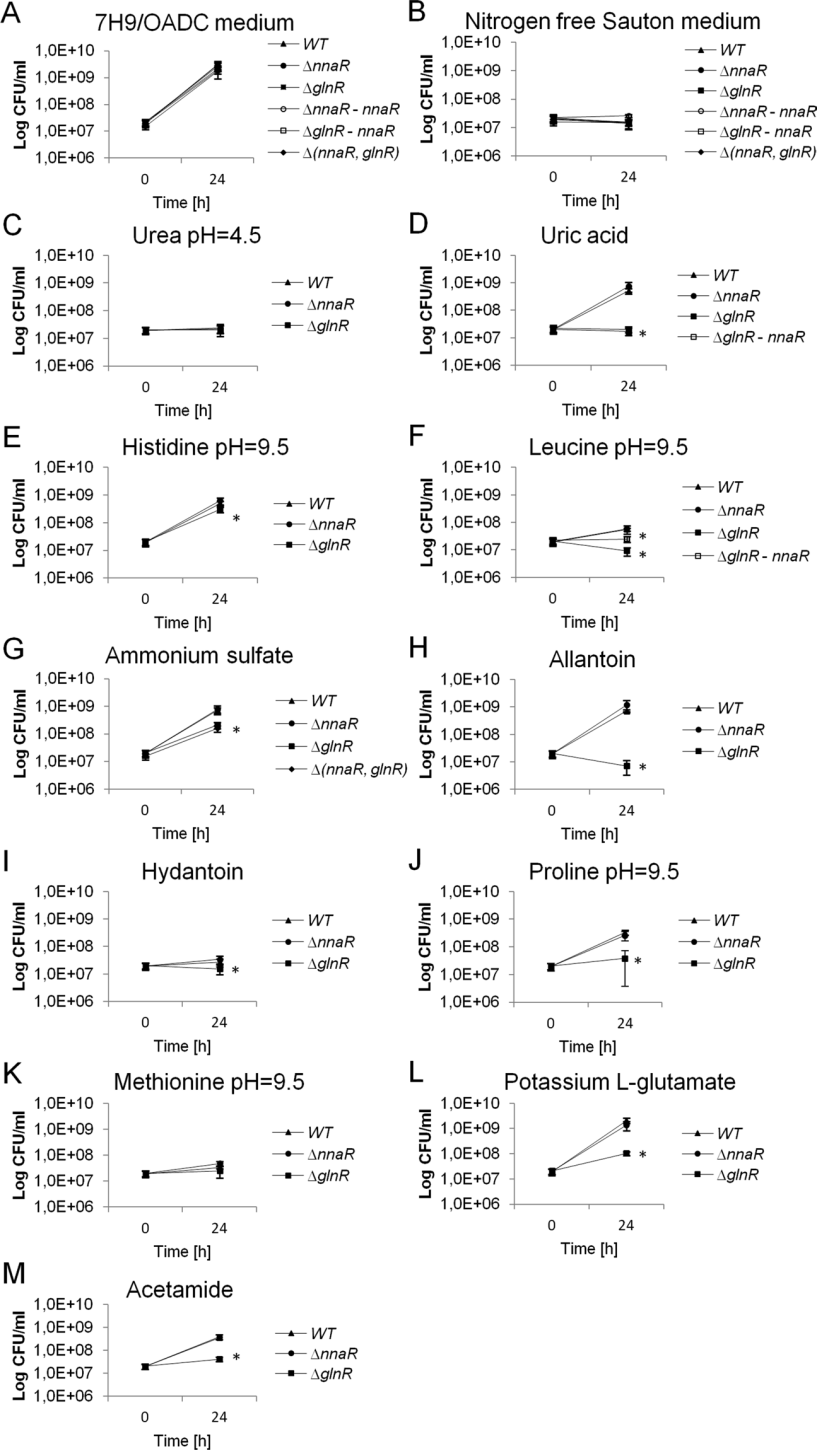
The survival of *M*. *smegmatis* strains propagated on nitrogen-limiting Sauton’s medium containing various nitrogen sources. Wild-type, ∆*nnaR*, ∆*glnR*, ∆*nnaR–attB::*_*phsp*_*nnaR*, ∆*glnR- attB::*_*phsp*_*nnaR* and ∆(*nnaR*, *glnR*) were grown in the presence of the (**C**) urea (pH = 4.5), (**D**) uric acid, (**E**) histidine (pH = 9.5), (**F**) leucine, (**G**) ammonium sulphate, (**H**) allantoin, (**I**) hydantoin, (**J**) proline, (**K**) methionine, (**L**) L-glutamic acid potassium salt monohydrate (each at 10 mM final concentration) and (**M**) acetamide (5 mM). Standard (**A**) 7H9/OADC medium was used as a positive control, and (**B**) nitrogen-free Sauton’s medium was the negative control. The numbers of viable cells were determined by counting of the bacterial colony-forming units (CFU) on 7H10/OADC plates at 24 hours. Colony formation values are means ± standard deviation from three independent experiments. The statistical significance was determined using Student’s t-test (*p < 0.03): (**D)** for ∆*glnR*, ∆*glnR– attB::*_*phsp*_*nnaR p* < 0.001, (**E**) for ∆*glnR p* < 0.001, (**F)** for ∆*glnR*, ∆*glnR– attB::*_*phsp*_*nnaR p* < 0.001, (**G**) for ∆*glnR p* < 0.001, (**H**) for ∆*glnR p* < 0.001, (**I**) for ∆*glnR p* = 0.014, (**J**) for ∆*glnR p* < 0.001, (**L**) for ∆*glnR p* < 0.001, (**M**) for ∆*glnR p* < 0.001.

The high-throughput transposon mutagenesis assays have revealed *nnaR* (*rv0260c*) is a non-essential gene in *M*. *tuberculosis*^18^; *nnaR* has been disrupted using transposon mutagenesis technique in *S*. *coelicolor*^14^. To evaluate the function of NnaR in *M*. *smegmatis* cells, we created a deletion strain using a protocol for homologous recombination^19^. The genotype of the obtained double-crossover (DCO) recombinants carrying the truncated, out-of-frame copy of the *nnaR* gene (Δ*nnaR)* was confirmed by PCR (data not shown) and Southern blot hybridization (Supplementary Fig. S1A,B). We analogously generated an *M*. *smegmatis* mutant strain lacking a functional copy of *glnR* (Δ*glnR*) (Supplementary Fig. S1C,D) and a double mutant lacking both genes *nnaR* and *glnR* Δ(*nnaR*, *glnR*). The Δ*nnaR-attB::*_*p*hsp60_*nnaR* and Δ*glnR-attB*::_*p*hsp60_*nnaR* complementation strains carrying an integration plasmid expressing *nnaR* copy under heat shock promoter (_*phsp*_) as well as Δ*glnR-attB*:: _*p*glnR_*glnR* complementation strain possessing *glnR* copy under its own promoter were also prepared. The primers and recombinant plasmids used in this study are listed in Supplementary Table S1. We analysed the kinetics of growth by measuring the OD_600_ (Supplementary Fig. S2A) and viability (CFU/mL) (Fig. 1A), which revealed no significant differences in growth or survival between tested mutant strains (Δ*nnaR*; Δ*glnR*; Δ(*nnaR*, *glnR*); Δ*nnaR-attB*::_*p*hsp60_*nnaR*; Δ*glnR-attB*::_*p*hsp60_*nnaR*) and wild-type when propagated in 7H9/OADC liquid media (nitrogen-rich environment).

### *M*. *smegmatis* Δ*nnaR* and Δ*glnR* mutant strains are defective in assimilation of various nitrogen sources according to phenotype microarray profiling

The mutants defective in the synthesis of NnaR or GlnR were subjected to phenotypic analysis using the BIOLOG Phenotype Microarray screening platform, which allows for convenient testing of growth kinetics of bacteria under various conditions. We exploited 6 PM plates (PM3-8) representing 576 different growth conditions, including various nitrogen sources, phosphorus and sulphur sources, nutrient supplements, and peptide nitrogen sources for simultaneous testing.

When comparing the area under the curve (AUC) values of the wild-type *M*. *smegmatis* and the Δ*nnaR* and Δ*glnR* mutants, several changes were noted in the assimilation of various nitrogen sources (Table S2). The mutant defective in the synthesis of NnaR was significantly less metabolically active than wild-type in the media containing guanosine (AUC difference >9,500), nitrite (>8,500), γ-amino-N-butyric acid (>7,500) or nitrate (>6,500). The list of significant differences between the mutant and wild-type strain was much longer in the case of Δ*glnR*. The mutant defective in the synthesis of GlnR was significantly less metabolically active than wild-type in the media containing various peptides and amino acids as nitrogen sources (approximately 100 hits with AUC differences between 7,000 and 30,000). The kinetics of Δ*glnR* metabolism was also affected in the presence of, e.g., uridine (>16,500), N-acetyl-D-glucosamine, D,L-α-amino-N-butyric acid (>12,500), uracil (>12,000), nitrite (>10,000) or nitrate (>7500). On the other hand, the Δ*glnR* mutant was more active than the wild-type in media supplemented with xanthine (>20,000), glucuronamide (>15,000), parabanic acid (>14,000), formamide (>12,000) or ethanolamine (>11,000) as nitrogen sources (Supplementary Table S2).

### Utilization of various nitrogen sources via the Δ*nnaR* and Δ*glnR* strains

The evidence from the global BIOLOG analysis prompted us to determine the growth and viability of the studied *M*. *smegmatis* strains in nitrogen-limiting medium containing various substances as the sole nitrogen sources. Most of the studied nitrogen compounds, such as urea (pH = 4.5), uric acid, histidine (pH = 9.5), leucine (pH = 9.5), proline (pH = 9.5), methionine (pH = 9.5), allantoin and potassium L-glutamate, were selected based on BIOLOG Phenotype Microarray analysis. We additionally tested the ability of the Δ*nnaR* strain to assimilate nitrogen from ammonium sulphate, hydantoin and acetamide. The kinetics of the growth and number of viable cells were evaluated in the presence of the tested nitrogen sources, following the induction of the initial nitrogen starvation to reduce background interference. The CFU analysis did not indicate significant differences in viability of Δ*nnaR* mutant cells in comparison to wild-type in the presence of the selected nitrogen-containing compounds. The Δ*nnaR* strain efficiently incorporated nitrogen derived from a majority of tested compounds, except for urea, hydantoin and methionine. In contrast, Δ*glnR* had significantly reduced survival in the presence of uric acid (96.6%), histidine (40.2%), leucine (83.5%), ammonium sulphate (71.3%), allantoin (99.0%), hydantoin (42.9%), proline (88.4%), potassium L-glutamate (95.1%) and acetamide (88,5%) in comparison to wild-type. Urea, methionine and hydantoin were the worst at supporting the growth of all studied strains when used as sole nitrogen sources (Fig. 1). The obtained viability results (CFU counts) were consistent with the measurements of the absorbance at 600 nm at the indicated time points (Supplementary Fig. S2). The presented viability data for Δ*glnR* mutants were statistically significant according to Student’s *t*-test (run in GraphPad software). The CFU data for Δ*nnaR* and Δ*glnR* were consistent with the previously observed phenotype microarray screening platform results for a majority of chosen nitrogen sources, except urea, allantoin and proline (Δ*nnaR*) or urea, leucine, allantoin and glutamate (Δ*glnR*). The survival assays were also in line with the previously published evidence, regarding the role of GlnR as the main nitrogen metabolism regulator. The kinetics of growth and viability have also been tested for Δ(*nnaR*, *glnR*) double mutant in liquid media containing ammonium sulphate as a sole nitrogen source. The optical density readings and CFU counts for the double mutant were nearly identical to those obtained for Δ*glnR* mutant (Fig. 1, Supplementary Fig. S2).

### Transcriptome profiling reveals NnaR functions in the regulation of nitrate/nitrate transport and utilization systems

Total RNA sequencing was employed to establish the NnaR regulon in *M*. *smegmatis*. The RNA profiles were compared between the wild-type, Δ*nnaR* and Δ*glnR* strains cultured in the defined Sauton’s minimal medium containing high levels of nitrogen (30 mM ammonium sulfphate) or under nitrogen-limiting conditions (1 mM ammonium sulphate). When comparing Δ*nnaR* to the wild-type strain, there were no significant differences between their transcriptomes when strains were cultured in nitrogen-rich media. Upon nitrogen depletion, a total of seven transcripts (corresponding to four single ORFs and three operons) were down-regulated in the mutant strain. In the wild-type strain, all these transcripts were induced specifically during nitrogen starvation, and their relative expression was low in nitrogen-rich growth medium. The proteins encoded by the down-regulated transcripts were involved in nitrogen utilization pathways. They included nitrate, nitrite and nucleotide-derived nitrogen cycle elements, as well as proteins implicated in nitrogen storage. One of the identified targets was the gene encoding bacterial truncated haemoglobin (MSMEG_5765, trHbN). MEME analysis of the promoter regions for the abovementioned transcripts revealed the presence of a partially degenerated palindromic motif (c-t-C-A-C-a/c-(16N)-t/g-G-T-G-a-g) located within the vicinity of the GlnR-binding motif (Supplementary Fig. S3). The two regulatory DNA motifs were spaced similarly in the majority of the transcripts. In the case of *msmeg_4008*, the spacing between the palindromic sequences was uneven, lacking a single nucleobase. Thus, it was only possible to assign two half-motifs, instead of the abovementioned motif. Consistently, the expression levels of all the seven transcripts were even more profoundly depleted in the Δ*glnR* strain. In line with the previously published microarray data, the Δ*glnR* strain showed a high number of transcriptional changes upon nitrogen depletion, as well as when grown on nitrogen-rich media (Supplementary Table S3). When cultured under nitrogen-limiting conditions, 537 individual genes (approximately 296 transcripts, counting operons as single transcripts) were up- or down-regulated by more than a log2 fold-change value of ±1.5. A total of 42 previously annotated GlnR motifs (out of 53)^3^ were associated with significant changes in gene expression under the conditions tested. The removal of *glnR* also led to a profound down-regulation of *nnaR* expression, making this strain a natural Δ*nnaR* mutant. The removal of *glnR* led to a transcriptional change of 633 genes when the mutant strain was grown on the nitrogen-rich medium. However, a different set of genes changed on the nitrogen-rich medium, and only 12 GlnR motifs were identifiable in front of the affected transcripts. Importantly, the differences observed between nitrogen-rich and nitrogen starvation growth conditions were much less sharp for the elements encoding nitrogen utilization enzymes, e.g., the log2 fold change of −2.33 versus −9.37 for the *nnaR* transcript. In fact, multiple nitrogen metabolism genes remained relatively unaffected, e.g., nitrite reductase (*msmeg_0427*, *msmeg_0428*) and nitrite extrusion protein (*msmeg_0433*), as they were not up-regulated under nitrogen-rich growth conditions and were rather associated with nitrogen starvation.

### Validation of RNA-Seq results by quantitative qRT-PCR and electrophoretic mobility shift assay (EMSA)

**Figure 2:**
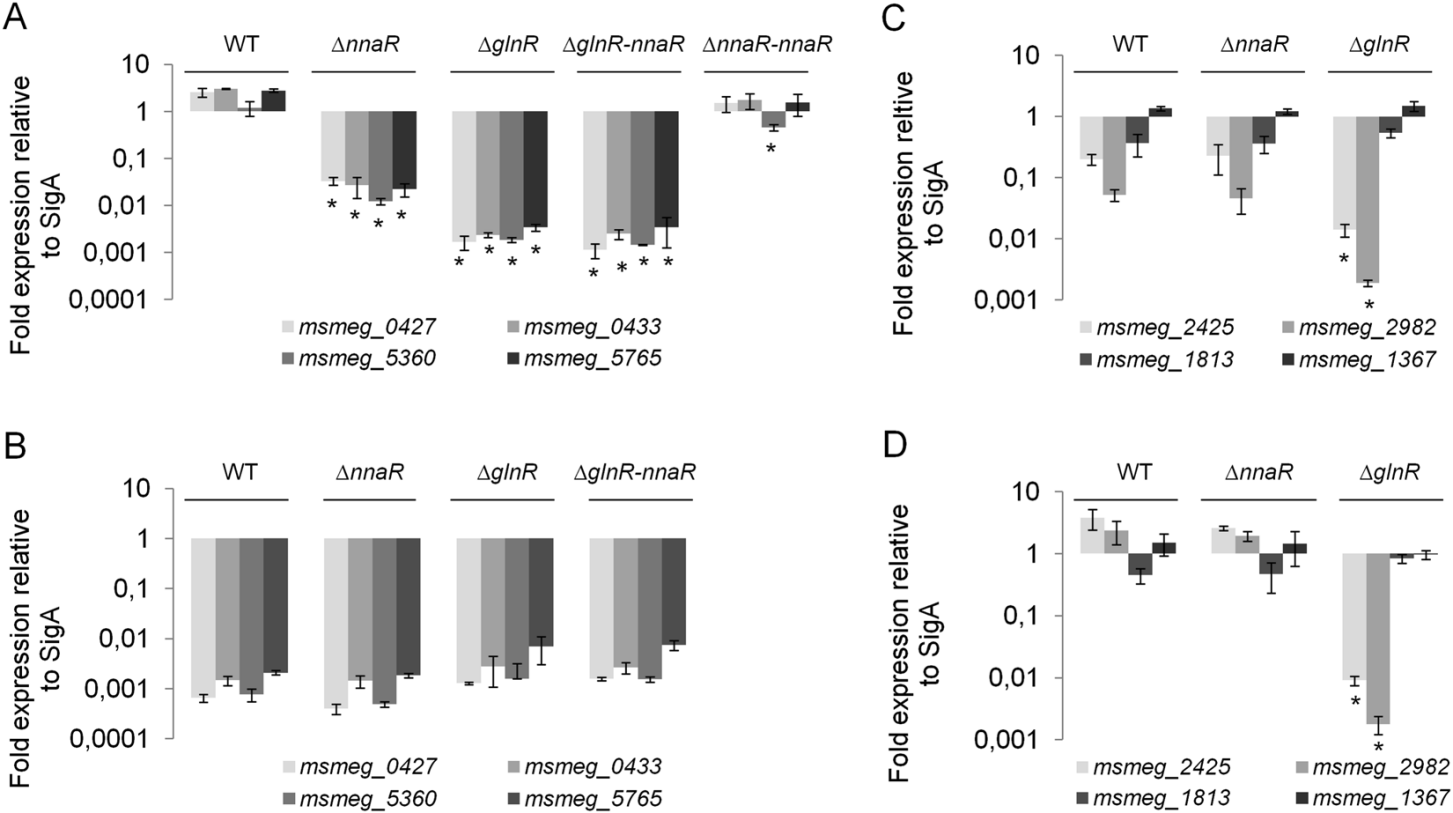
Expression profiles of select genes in ∆*nnaR*, ∆*glnR* mutant strains and ∆*glnR–attB::*_*phsp*_*nnaR*, ∆*nnaR–attB::*_*phsp*_*nnaR* complementing strains. RNA samples prepared from *M*. *smegmatis* cultures growing at nitrogen depletion **(A**,**B)** or nitrogen-rich environment **(C**,**D)** were processed for evaluation of expression for: *msmeg_0427*, *msmeg_0433*, *msmeg_5360*, *msmeg_5765*, *msmeg_2425*, *msmeg_2982*, *msmeg_1813* and *msmeg_1367*. The qRT-PCR reactions were performed using SYBR green chemistry and data are normalized to *sigA* rRNA levels. Mean values and standard deviations from three independent experiments are shown. The statistical significance was determined using Student’s t-test (*p < 0.01).

To validate the expression profiles obtained by RNA-Seq, qRT-PCR was performed on eight chosen transcripts that enabled us to differentiate between the wild-type and the investigated mutant strains cultured under nitrogen starvation or nitrogen-rich conditions. We selected four genes that had a reduced level of expression in the Δ*nnaR* and Δ*glnR* mutant strains: *msmeg_0427* (*nirB*, nitrate reductase), *msmeg_0433* (*narK*, nitrate extrusion protein), *msmeg_5360* (formate/nitrate transporter), *msmeg_5765* (truncated bacterial haemoglobin, *trHbN*); we also selected two genes that had very reduced expression in Δ*glnR* compared to Δ*nnaR* and that are involved in ammonia and urea transport, respectively: *msmeg_2425* or *msmeg*_2982. As a control, we selected two genes unrelated to nitrogen metabolism: the gene encoding AccD5 carboxyltransferase (*msmeg_1813*), involved in cell envelope lipid biosynthesis^20^, and *rpoB* (*msmeg_1367*), encoding the β-subunit of the RNA polymerase^21^. The same RNA samples as extracted for library preparation were used for qRT-PCR validation. qRT-PCR analyses confirmed the results obtained from RNA-Seq. Under nitrogen depletion, the transcript levels of *msmeg_0427*, *msmeg_0433*, *msmeg_5360*, and *msmeg_5765* from *M*. *smegmatis* Δ*nnaR* and Δ*glnR* mutants were down-regulated compared to wild-type. The levels of tested here transcripts remained low in Δ*glnR* strain complemented with *nnaR* (Δ*glnR*-*attB::*_*p*hsp60_*nnaR*). On the other hand, the same set of transcripts returned to wild-type levels for Δ*nnaR* complemented *nnaR* (Δ*nnaR-attB*::_*p*hsp60_*nnaR*) (Fig. 2A). The collected data revealed no significant changes in the expression levels in the above genes for Δ*nnaR*, Δ*glnR*, Δ*glnR*-*attB::*_*p*hsp60_*nnaR* and the wild-type strain, under nitrogen-rich environment (Fig. 2B). The qRT-PCR analyses confirmed lower expression of *msmeg_2425* (1.32 log10 fold change (FC)) and *msmeg_2982* (1.48 log10 FC) in Δ*glnR* in comparison to Δ*nnaR* under nitrogen depletion (Fig. 2C) and in rich environment: *msmeg_2425* (2.50 log10 FC), *msmeg_2982* (2.99 log10 FC) (Fig. 2D). As expected, no significant changes in *accD5* or *rpoB* were observed in either studied condition (Fig. 2C,D).

**Figure 3:**
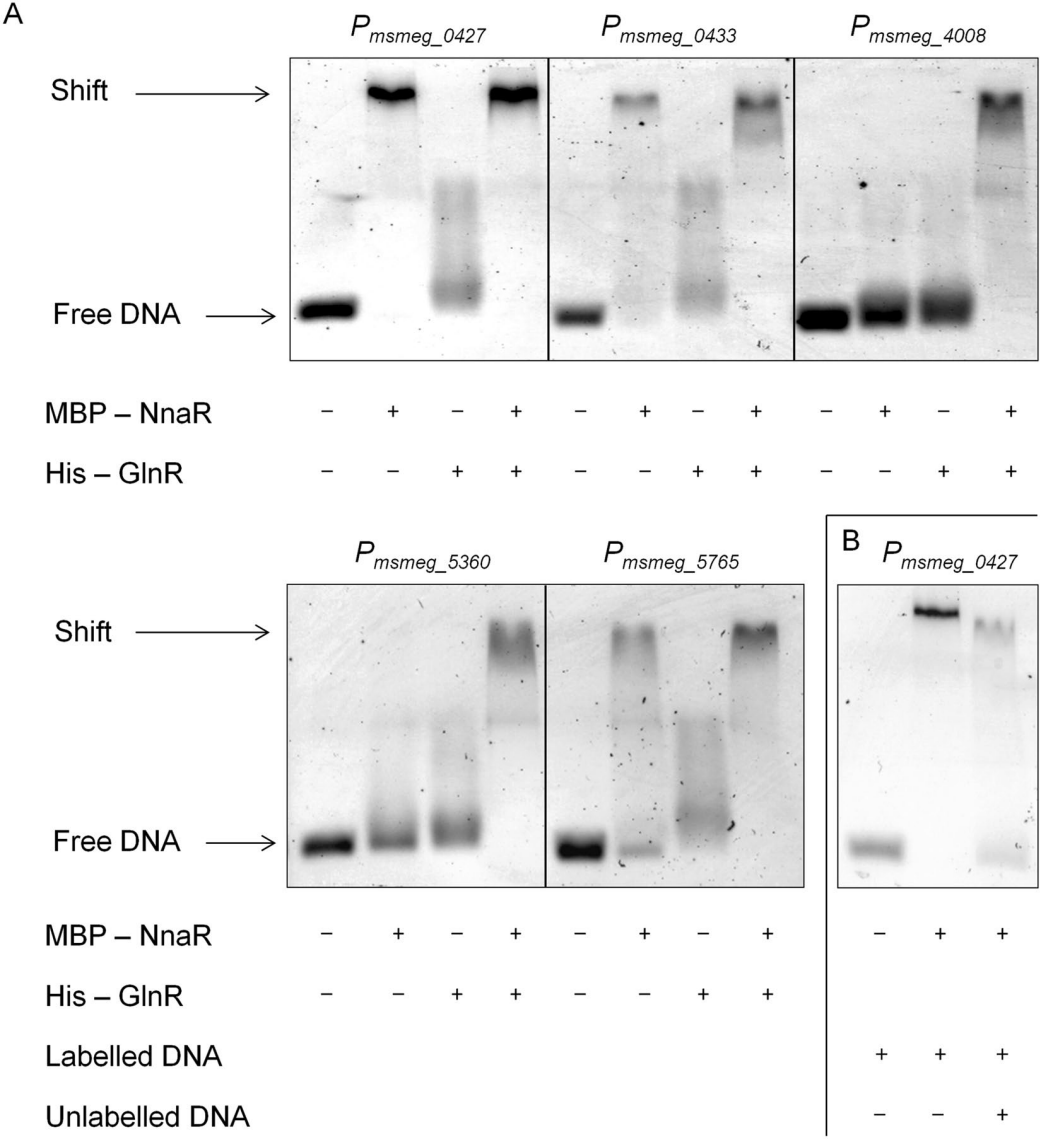
Interactions between NnaR, GlnR or NnaR/GlnR mixture and putative promoter regions of *msmeg_0427*, *msmeg_0433*, *msmeg_4008*, *msmeg_5360* and *msmeg_5765* analysed by EMSA. For EMSA reactions (**A**), approximately 30 nM hexachlorofluorescein-labelled DNA was incubated with 0 or 2 µM NnaR, 4 µM GlnR or 2 µM NnaR combined with 4 µM GlnR for 10 min in EMSA reaction buffer containing 10 mM Tris, 100 mM KCl, 1 mM DTT, 2.5% glycerol, 20 mM MgCl_2_, 500 ng poly(dI·dC), and 0.05% NP-40 (pH 7.5). (**B**) NnaR binding specificity to the promoter region of *msmeg_0427* was analysed using the competition test. Approximately 30 nM hexachlorofluorescein-labelled DNA or a combination of both 30 nM labelled and 100-fold excess unlabelled probe was incubated with 2 µM NnaR for 10 min in EMSA reaction buffer. The samples were resolved in 2% agarose gel and visualized on a GE Typhoon 8600 Imager.

The EMSA assay was applied to further facilitate the validation of RNA-Seq results and to cross-confirm the NnaR-specific DNA-binding motif identified via MEME search. To show a direct regulation of identified genes by NnaR and GlnR, we purified the recombinant versions of both response regulators to assess their DNA-binding properties. The NnaR protein was purified as a full-length polypeptide possessing both the uroporphyrinogen-III synthetase and DNA-binding domains and tagged with maltose-binding protein (MBP) at the N-terminus. The GlnR protein was purified as a fusion with an N-terminal poly-histidine (His) tag. The putative promoter sequences of *msmeg_0427*, *msmeg_0433*, *msmeg_4008*, *msmeg_5360* and *msmeg_5765* were amplified using hexachlorofluorescein-labelled primers to visualize the DNA fragments after electrophoresis under native conditions. All of the DNA fragments (with the exception of the sequence upstream the *msmeg_4008* gene) contained the putative NnaR motif. The NnaR protein bound avidly to the putative promoters of *msmeg_0427*, *msmeg_0433* and *msmeg_5765* but did not recognize the sequence upstream of *msmeg_4008* or *msmeg_5360* at 2 µM NnaR concentration (Fig. 3A). On the other hand, modest interaction was observed for GlnR with *msmeg_0427*, *msmeg_0433* and *msmeg_5765* promoter sequences but not with *msmeg_4008* and *msmeg_5360* promoters. Interestingly, the mobility of oligos representing *msmeg_4008* and *msmeg_5360* promoters was affected in the presence of both investigated proteins applied simultaneously, indicating possible cooperation of NnaR and GlnR (Fig. 3A). To test if the proteins may act as a stable protein complex we have performed a pull-down experiment with recombinant response regulators used for EMSA. We could only observe weak association between the two proteins (Supplementary Fig. S4) and they failed to interact with each other when tested in the bacterial two hybrid method (data not shown).

To further confirm the specificity of the NnaR protein towards the tested sequences, we also carried out a competition experiment. The NnaR protein caused a shift of the entire labelled *msmeg_0427* DNA fragment when it was incubated with the sole labelled probe. The binding was nearly abolished in the presence of a competitor DNA fragment used at 100-fold excess relative to the labelled probe (Fig. 3B).

### The Δ*nnaR* and Δ*glnR* mutant strains show severe growth defects on nitrate and nitrite

**Figure 4:**
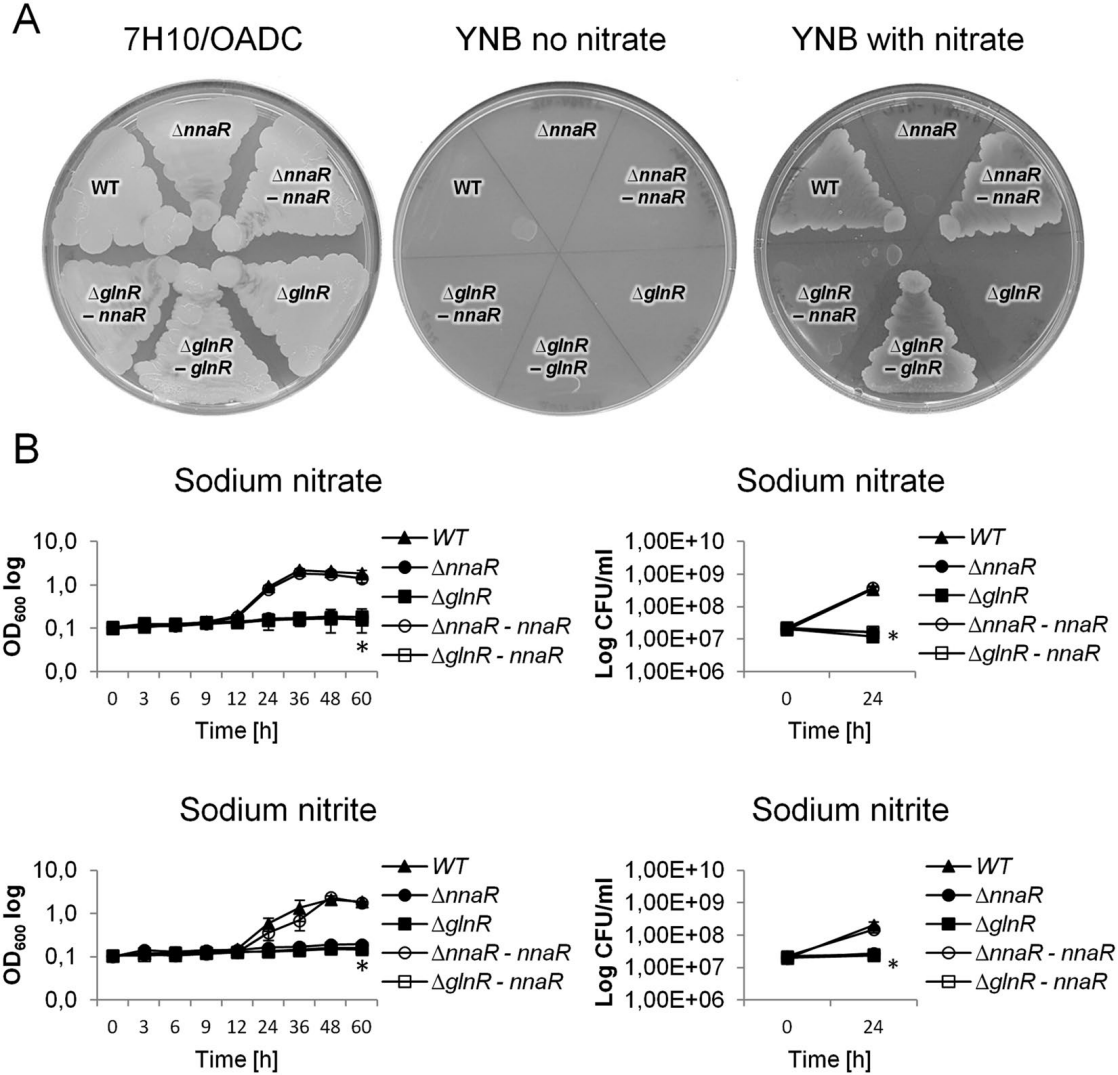
Growth of the wild-type and ∆*nnaR*, ∆*glnR*, ∆*nnaR–attB::*_*phsp*_*nnaR*, ∆*glnR-attB::*_*p*glnR_*glnR M*. *smegmatis* strains on solid medium: Middlebrook 7H10/OADC, Yeast Nitrogen Base Agar without nitrogen source and Yeast Nitrogen Base Agar with sodium nitrate (10 mM) **(A)**. The images are examples of three biologically independent experiments. **(B)** Growth kinetics and survival of the wild-type and ∆*nnaR*, ∆*glnR*, ∆*nnaR–attB::*_*phsp*_*nnaR*, ∆*glnR-attB::*_*phsp*_*nnaR M*. *smegmatis* strains on nitrogen-limiting Sauton’s medium containing sodium nitrate (10 mM) and sodium nitrite (5 mM). The kinetics of growth were evaluated by measuring the absorbance at 600 nm at the indicated time points. The number of viable cells at 24 hours was counted by the CFU method. The displayed values are the average ± standard deviation from three independent experiments. The statistical significance was determined using Student’s *t*-test (*p < 0.03): for ∆*nnaR*, ∆*glnR*, ∆*glnR–attB::*_*phsp*_*nnaR p* < 0.001 (CFU on sodium nitrate), for ∆*nnaR*, ∆*glnR*, ∆*glnR–attB::*_*phsp*_*nnaR p* < 0.001 (CFU on sodium nitrite).

The collective evidence from the global RNA-Seq and high-throughput BIOLOG Phenotype screening platforms indicated that NnaR functions as a nitrogen assimilation regulator in mycobacteria. To evaluate the importance of NnaR protein in nitrogen metabolism/assimilation in *M*. *smegmatis* cells, we examined the growth of Δ*nnaR* mutant and Δ*nnaR*-*attB::*_*phsp60*_*nnaR* complementation strain on nitrogen-limiting agar, YNB, containing sodium nitrate as sole nitrogen source (later referred to as “YNB-nitrate”). We used Δ*glnR*, Δ*glnR*-*attB*::_*p*glnR_*glnR* complementation strain and wild-type strain as controls. Full growth for all tested strains was observed on nitrogen-rich agar plates (7H10/OADC). The Δ*nnaR* and Δ*glnR* cells did not exhibit any growth on YNB-nitrate plates, while the Δ*nnaR-attB*::_*p*hsp60_*nnaR*, Δ*glnR*-*attB*:: _*p*glnR_*glnR* complementation strains and wild-type cells showed full growth on nitrogen-limiting medium (Fig. 4A).

To further confirm the above results, we examined the kinetics of growth and viability of the Δ*nnaR* mutant and the Δ*nnaR-attB*::_*p*hsp60_*nnaR* complementation strain in the presence of nitrate (sodium nitrate) and nitrite (sodium nitrite) as the sole source of nitrogen. The Δ*glnR* mutant and the Δ*glnR* strain complemented with an additional copy of *nnaR*, controlled by a heat shock promoter, as well as wild-type, were the additional controls in this experiment. Growth experiments for all tested strains were carried out following a 16 hours of nitrogen starvation to deplete the intracellular pool of nitrogen stored inside the cells. A significant reduction in the growth kinetics and viability of Δ*nnaR* and Δ*glnR* cells was observed compared to wild-type. The mutants had completely lost their ability to assimilate both nitrate and nitrite, which could be explained by the RNA-Seq results (Fig. 4B). After 24 hours of growth on the sodium nitrate-containing media, the viability of Δ*nnaR* cells was reduced by approximately 95% compared to the wild-type strain. Supplementation of the culture media with sodium nitrite resulted in a reduction in survival of Δ*nnaR* cells by approximately 87%. The Δ*nnaR-attB*::_*p*hsp60_*nnaR* complementation strain reversed the observed phenotype back to the wild-type phenotype. Similarly to Δ*nnaR*, the Δ*glnR* mutant cells grown on nitrate and nitrite as the sole source, exhibited significant reduction in viability in comparison to the wild-type strain (96.5% or 88.5%, on nitrate and nitrite, respectively) (Fig. 4B). Interestingly, the survival of Δ*glnR* mutant cells was not rescued when a complementing copy of *nnaR* was introduced to this mutant. Hence, NnaR protein was not able to compensate for the lack of GlnR. The observed results suggest that both response regulators are required for nitrate/nitrite assimilation.

### The ability of Δ*nnaR* and Δ*glnR* of *M*. *smegmatis* to convert nitrate to nitrite and nitrite to ammonia

**Figure 5:**
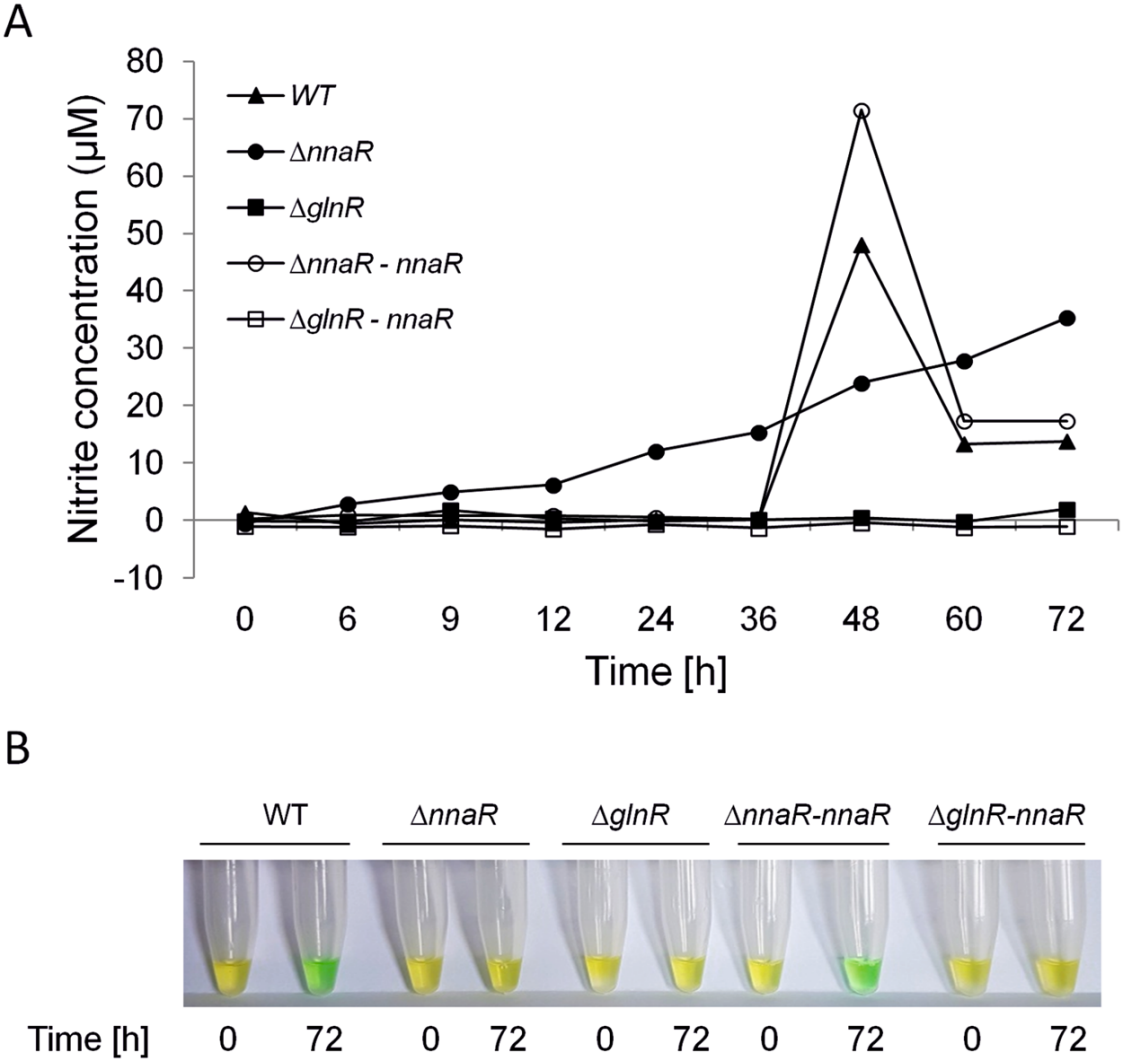
The ability of ∆*nnaR*, ∆*glnR*, ∆*nnaR–attB::*_*phsp*_*nnaR*, ∆*glnR-attB::*_*phsp*_*nnaR M*. *smegmatis* strains to reduce nitrate to nitrite and nitrite to ammonium. **(A)** *M*. *smegmatis* cells were grown on nitrogen-limiting Sauton’s medium containing 10 mM sodium nitrate as sole nitrogen source. Accumulation of nitrite at the indicated time points was assessed by measuring the nitrite concentration using the Griess Reagent System Kit. Three biologically independent experiments were performed, and a representative graph is shown. (**B**) The presence of ammonium ions in the same set of samples was assessed at the indicated time points using the ammonium test (Merck). The samples were tested according to the manufacturer’s protocol, while the reactions were downscaled accordingly. The presence of green colour, resulting from the formation of indophenol blue derivative, indicates the presence of ammonium ions in the culture’s medium. Representative samples are shown.

To determine whether the observed reduction in survival of Δ*nnaR* cells was due to the inability of the mutant cells to convert nitrate to nitrite, the Griess Reagent System was applied following the manufacturer’s instructions. Measurement of the nitrite in the supernatants obtained after centrifugation of bacterial cells from the medium containing sodium nitrate as the sole source of nitrogen revealed that Δ*nnaR* reduced nitrate to nitrite, but it could not assimilate the product of this conversion (Fig. 5A). Compared to the wild-type strain, the growth of the mutant was not observed, but the increase in the amount of nitrite (as a nitrate conversion product) in growth medium was noticeable. The restoration of the functional NnaR in the cell, as in the NnaR complementation strain, restored the ability of the bacterium to incorporate nitrite into the intracellular biomolecules. This observation suggests that the nitrate reduction pathway is at least partially active in Δ*nnaR* and other factors are responsible for the observed loss of viability. From previous work in *Streptomyces*^14^ and based on the RNA-Seq data, it seemed that *nirBD* expression may be the essential factor providing bacterial survival when nitrate/nitrite is the only available source of nitrogen. Thus, we tested the presence of ammonium ions in the same samples by using a colorimetric method. Confirming our hypothesis, the ammonium ion was detectable in the supernatants from cultures of the wild-type and the Δ*nnaR-attB*::_*p*hsp60_*nnaR* strains at 72 hours (Fig. 5B). The concentration of ammonium remained at the background level in the culture media from the NnaR mutant strain. In contrast, Δ*glnR* could not reduce nitrate to nitrite, which is consistent with the complete lack of growth of this strain in the presence of sodium nitrate. The introduction of an intact copy of the *nnaR* gene under the highly active *hsp60* promoter into Δ*glnR* mutant did not restore the ability of that strain to convert nitrate to nitrite, suggesting both transcriptional regulators need to be active for the effective expression of nitrogen-converting enzymes (Fig. 5A).

### Biofilm formation of *M*. *smegmatis* Δ*nnaR* mutant strain

It has been observed that *glnR* response regulator is upregulated during biofilm formation of *M*. *smegmatis*^22^. In a further study, Yang *et al*. has observed delayed biofilm development of Δ*glnR* as well as GlnR-dependent resistance to hydrogen peroxide under nitrogen limiting conditions^23^. To test whether the removal of NnaR affects biofilm development of *M*. *smegmatis* we have examined the growth of Δ*nnaR* strain on Sauton’s_rich_, Sauton’s_N1/2_ and Sauton’s_N0_ media^23^, in the absence of any detergents. We compared the growth of Δ*nnaR* strain to wild-type as well as Δ*glnR* strains as controls for our experiment. While we have observed a delay in biofilm formation for Δ*glnR*, as reported by Yang and colleagues^23^, we were unable to note any significant changes in the kinetics of biofilm formation of Δ*nnaR* strain, which behaved similarly to the wild-type strain (Supplementary Fig. S5). None of the strains was able to produce biofilm in Sauton’s nitrogen-free medium (data not shown).

## Discussion

The GlnR protein is currently considered the key response regulator contributing to the survival of mycobacterial cells during nitrogen starvation^2–5,7,10^. The gene encoding GlnR is not essential when mycobacteria reside within nitrogen-rich niches, but it becomes indispensable under nutrient-limiting conditions^5^. The regulatory DNA motifs are fairly well characterized for the GlnR protein from *M*. *smegmatis* and *M*. *tuberculosis*, as well as some other actinomycetes, showing good conservation among different species^3,5,24,25^. During our analysis, we discovered that there was also a great level of similarity between the regulatory motifs recognized by the GlnR factor and another “global” response regulator, MtrA^26,27^. The motif consensus and the internal spacing between the motif residues are very well conserved, with nearly identical sequences. Corroborating this, we discovered that the DNA binding domain of MtrA and GlnR were nearly identical, and both transcriptional regulators were thus likely to bind highly similar motifs (Supplementary Fig. S3C). On the other hand, MtrA was previously shown to bind the promoter sequence of the *nirB* gene in mycobacteria^28^. Another study found GlnR competed with PhoP to bind promoter regions of the *glnA* and *amtB* genes in *S*. *coelicolor*^24^. The regulatory networks require an interplay between all the response regulators acting in a highly orchestrated way to maximize the adaptive responses of the bacterium. On the other hand, one gene’s expression may be regulated by a number of transcription factors, and they may play either synergistic or antagonistic roles in the regulation of gene expression. The complicated dependencies between various factors contributing to the transcriptional regulation of a given gene are not very well understood.

The GlnR regulon is fairly well characterized for some actinomycetes, with the response regulator acting mainly as a transcriptional activator during nitrogen starvation and less often playing a role of transcription repressor^29,30^. Using the CHIP-Seq approach, a previous study identified approximately 53 GlnR boxes on the *M*. *smegmatis* genome, most of them potentially being regulatory motifs^3^. The same study identified a much wider set of genes with altered expression in the absence of GlnR using the microarray approach. Another study used the same model organism to discover that some additional transcripts changed significantly in the response to nitrogen depletion on the Δ*glnR* background, also using microarray technology^31^.

Among the multiple genes regulated directly by the activity of GlnR are transcription factors, adding to the overall complexity of the GlnR dependent response. One such transcription factors is NnaR, belonging to the same class of response regulators of the two-component systems family^14^. Both GlnR and NnaR are considered orphan response elements, lacking any identifiable, genetically linked histidine kinase. A report from *Streptomyces* revealed a potential role of serine/threonine phosphorylation and some lysine acetylation among the mechanisms by which the activity of GlnR protein is regulated^17^. On the other hand, GlnR controls genes encoding lysine deacetylases in Actinobacteria^32^. Such mechanisms may further alter the ability of the protein to bind DNA, modulating its DNA-binding strength and/or substrate specificity, making GlnR an intriguing model to study. Another *Streptomyces* study focused on elucidating the activity of NnaR protein, a regulator activating expression of a small set of genes critical for nitrate/nitrite assimilation. Based on *in silico* analyses, the study identified a potential NnaR-binding motif in the vicinity of the GlnR binding motifs, present in front of a nitrite reductase, NirB, a putative nitrate reductase, NasA, and the NarK nitrate/nitrite transporter^14^. To investigate the activity of NnaR in mycobacterial species, we used the *M*. *smegmatis* as the model. Since NnaR expression is directly affected by the activity of GlnR, we also intended to study their interconnection in more detail.

We initially generated unmarked mutant strains lacking the NnaR or the GlnR orphan response regulator via the standard two-step recombination protocol^19^. We next subjected such mutant strains to global analyses using BIOLOG Phenotype Microarray screen^33^ and transcriptomic profiling using total RNA sequencing. Using the two independent platforms, we detected nitrate/nitrite metabolism as the key pathway regulated by the activity of NnaR. We performed the transcriptional profiling under the conditions previously reported for the GlnR profiling microarray experiments^3^, to be able to cross-validate our results. The removal of NnaR caused only subtle transcriptomic rearrangements, significantly changing the expression level of twenty genes (three operons and eight single open reading frames), but only when the strains were cultured under nitrogen-limiting conditions. Similar to what was predicted based on the *in silico* analysis from *Streptomyces*^14^, the affected transcripts included the nitrite reductase NirBD (*msmeg_0427*, *msmeg_0428*), the NarK transporter (*msmeg_0433*) and the pseudogene for NasA (*msmeg_4206*). We additionally identified *msmeg_5360*, a formate/nitrate transporter, as well as *msmeg_5765* as two more transcripts requiring the activity of NnaR for their expression under nitrogen depletion in *M*. *smegmatis*. Two additional operons, encoding genes mainly required for nucleotide transport and recycling, were affected by the removal of NnaR. Both operons encoded phenylhydantoinase genes, needed for the recycling of nitrogen from hydantoin. Thus, we tested the ability of the Δ*nnaR* mutant and wild-type strains to utilize hydantoin and allantoin as the sole source of nitrogen to support the growth of *M*. *smegmatis*. However, the laboratory strain of *M*. *smegmatis* failed to utilize hydantoin efficiently, showing only residual growth. It might have lost the full pathway necessary for the efficient utilization of such molecules as nitrogen sources. It is possible, however, that the environmental strains of mycobacteria use NnaR to control the expression of hydantoin-recycling factors, just as they express a full copy of the NasA nitrate reductase, which is only a pseudogene in the *M*. *smegmatis* MC2 155 (MSMEG) laboratory strain. We noted virtually no significant differences between the transcriptomes of the wild-type strain and the NnaR mutant grown in the presence of excess ammonium. The transcriptome profiling was followed by a wide screening of any potential phenotypic changes that may have resulted from the observed changes at the RNA level. Only nitrite and nitrate were selected as critical target molecules for the regulation of NnaR in the laboratory MSMEG strain. In our study, the Δ*nnaR* strain was completely unable to grow on nitrate or nitrite as sole nitrogen source, making the NnaR protein a conditionally essential gene for mycobacteria. The mechanism of bacterial killing relied most likely on the inability of the Δ*nnaR* strain to reduce nitrite to ammonia in the absence of sufficient expression of NirBD nitrite reductase. The Δ*nnaR* mutant showed no changes in growth, survival or transcriptomic profile when cultured on ammonium salts as nitrogen sources. Additional phenotypic alterations in response to NnaR inactivation cannot be ruled out and may be more pronounced in the environmental strains, with nucleotide scavenging and recycling being the most promising targets. This hypothesis was also confirmed by the BIOLOG screen, showing reduced growth of the Δ*nnaR* mutant on guanosine and γ-amino-N-butyric acid as nitrogen sources.

Interestingly, the NnaR protein contains an N-terminal HemD domain and was originally annotated as a bifunctional protein that was a putative uroporphyrinogen-III synthase and a response regulator. The abovementioned study on *Streptomyces* showed that NnaR does not possess HemD activity^14^. *M*. *smegmatis* possesses another HemD paralog, MSMEG_0954, which is likely the actual uroporphyrinogen-III synthase. Interestingly, NnaR is required to support the expression of the bacterial truncated haemoglobin, *trHbN* (*msmeg_5765*), which is one of the highest-affinity targets for NnaR-dependent expression. Studies on *M*. *tuberculosis*, *M*. *bovis* and *M*. *smegmatis* have shown that trHbN is involved in the adaptation to nitrite, acting as a nitrosative stress inducer. TrHbN together with another truncated haemoglobin, trHbO, contributes significantly to the survival of *M*. *tuberculosis* during macrophage infection, reducing the nitrosative and oxidative stress levels. TrHbN protects aerobic respiration from being inhibited by nitric oxide radical with its potent nitric oxide dioxygenase activity^34,35^. Studies on the *trHbN* promoters from *M*. *tuberculosis* and *M*. *smegmatis*^36,37^ suggest the presence of an iron/haeme-containing oxygen sensor that is involved in the modulation of the expression of truncated haemoglobins^36^. Taking into account our transcriptomic data and the presence of a HemD-like domain (possibly interacting with haeme or haeme-like molecules) in the N-terminus of NnaR, we are confident that NnaR is the regulator required for *trHbN* expression. This makes NnaR an important factor modulating the bacterial response to nitrosative stress, which is one of the key elements of the interplay between macrophages and *M*. *tuberculosis*, showing NnaR contributes to bacterial fitness and intracellular survival. The *trHbN* gene is listed among the elements of the GlnR regulon in *M*. *tuberculosis*^5^, likely requiring co-regulation of NnaR-GlnR to fully activate its transcription, similarly to what we can see in *M*. *smegmatis*.

All the transcriptomic changes caused by NnaR acting as a transcriptional activator were further deepened by the removal of GlnR. In other words, the NnaR regulon was even less efficiently expressed in the cells lacking GlnR. Adding another copy of NnaR under a strong promoter did not rescue the survival of the GlnR-deficient strain, suggesting that co-regulation rather than separate effects or the lack of NnaR expression on the Δ*glnR* mutant background were responsible for the observed phenomena. This notion was previously suggested by the abovementioned work on *Streptomyces’* NnaR protein^14^. We have collected additional pieces of evidence to support that finding. Importantly, we have noticed some more unique characteristics of the transcripts most profoundly affected by the removal of GlnR and NnaR. The main observation was that the spacing between the two motifs was fairly conserved for most of the studied transcripts, which carry features of leaderless transcripts. The transcription start site was located downstream of the GlnR-binding motif. These characteristics also applied to the promoter region of the *trHbN* gene, containing a putative GlnR box, downstream of the NnaR-binding motif. It is thus likely that the two response factors co-regulate the expression of the entire NnaR regulon. In our case, it is likely that NnaR acts as a sensing molecule, using an iron/haeme antenna to detect oxygen and nitrogen levels and serving as a proxy for GlnR-dependent transcription activation. One cannot rule out a NnaR-GlnR-like mode of co-regulation between GlnR, as a regulatory hub, and other types of response regulators in mycobacteria.

## Materials and Methods

### Strains and bacterial growth conditions

*Escherichia coli* strains were grown in Luria-Bertani broth (LB) or agar plates supplemented with ampicillin (50 μg/mL), kanamycin (50 μg/mL) or hygromycin (200 μg/mL). *M*. *smegmatis* strains were propagated in Middlebrook 7H9 (Difco Laboratories) liquid medium supplemented with 10% OADC (oleic acid, albumin, dextrose, catalase), 0.05% Tween 80 and kanamycin (25 μg/mL) or 7H10 medium supplemented with 10% OADC and 0.2% glycerol. For some experiments, such as culture preparation for RNA-Seq analysis and growth assays under nitrogen limitation, a defined nitrogen-free Sauton’s medium was applied (0.05% KH_2_PO_4_, 0.05% MgSO_4_, 0.2% citric acid, 0.005% ferric citrate, 0.2% glycerol, 0.0001% ZnSO_4_, 0,015% tyloxapol)^3^. To examine the growth of the studied strains on sodium nitrate as the sole source of nitrogen, 1.5% agar plates containing yeast nitrogen base medium lacking any amino acids and ammonium sulphate (YNB) supplemented with 10% AD were applied. All strains used in the study are listed in Table S1.

### Gene cloning strategies

All plasmid isolation, transformation and cloning techniques were performed essentially according to the protocols by Sambrook and Russell (2001)^38^. All PCR products were generated using thermostable AccuPrime*Pfx* DNA polymerase (Invitrogen) and cloned initially into a blunt vector (pJET 1.2/blunt; Thermo Fisher Scientific). Next, genes of interest were sequenced, released by digestion with appropriate restriction enzymes and cloned into the final vectors. The primers and plasmids used in this work are listed in Table S1.

### Construction of gene replacement vectors and complementation plasmids

Suicidal delivery vectors carrying deletion of *msmeg_0432* (*nnaR*) or *msmeg_5784* (*glnR*) were prepared in three steps. First, the 5′ upstream region of *nnaR* or *glnR* (1560 bp or 1484 bp, respectively) was cloned into a suicidal recombination delivery vector, p2NIL (Table S1). Next, a 1396 or 1660 bp fragment of *nnaR*’s or *glnR*’s downstream regions were ligated with plasmids from step 1 to create truncated, out-of-frame copies of the respective genes. Finally, a 6 kb PacI cassette from pGOAL17 was added, resulting in suicidal delivery vectors pMA3 and pRD152, used to engineer the directed *M*. *smegmatis* mutant strains.

To prepare the complementation plasmid, the *nnaR* gene was subcloned under the control of a heat shock promoter Hsp60 (Table S1) in the pMV261 vector to create pMA4. Next, a 1576 bp fragment consisting of *nnaR* and the Hsp60 promoter was released using XbaI/HindIII and cloned into pMV306K to create the final pMA5 construct. The obtained plasmid was integrated into the *attB* attachment site of the directed ∆*glnR* and ∆*nnaR M*. *smegmatis* mutants.

In order to generate a *glnR* complementation plasmid, the *glnR* gene (786 bp) and its putative promoter (500 bp) was PCR amplified, verified by sequencing and cloned in the integration vector pMV306 (pRD154), allowing for integration of the whole plasmid into a single –*attB* site on *M*. *smegmatis* chromosome. The resulting pRD154 plasmid was electroporated into Δ*glnR M*. *smegmatis* competent cells and the integration was confirmed by PCR.

### Disruption of *M*. *smegmatis nnaR* and *glnR* genes at their native chromosomal loci

A two-step protocol for homologous recombination^19^ was applied to generate defined mutant strains lacking functional NnaR or GlnR proteins as described previously^33,39^. The suicidal recombination plasmids pMA3 and pRD152 were treated with 0.2 mM NaOH and integrated into the *M*. *smegmatis* chromosome by homologous recombination. The obtained single-crossover (SCO) recombinants were blue, Kan^R^ and sensitive to sucrose. The site of recombination was confirmed by PCR and Southern hybridization (data not shown). The SCO strains were further processed to select for double-crossover (DCO) mutants that were white, Kan^S^ and resistant to sucrose (2%). The genotypes of the obtained mutant DCO strains were confirmed by Southern blot hybridization using the Amersham ECL Direct Nucleic Acid Labelling And Detection System (GE Healthcare) following the manufacturer’s instructions. Probes used were generated by PCR using primers listed in Table S1. In order to construct a double mutant strain, lacking functional copies of both *nnaR* and *glnR* genes, the suicidal delivery vector pRD152 carrying a Δ*glnR* gene was electroporated into Δ*nnaR M*. *smegmatis* competent cells. The SCO and further DCO screening was performed as described above. The genotype of the obtained double mutant strain Δ(*nnaR*, *glnR*) was confirmed by PCR and Southern hybridization using probe homologous to the 3′ of *glnR* gene (Supplementary Fig. S1).

### Cloning, expression and purification of NnaR and GlnR proteins

The *nnaR* (*msmeg_0432*) and *glnR* (*msmeg_5784*) coding regions were PCR-amplified using primers listed in Table S1. The *nnaR* gene was cloned into the pMALC4e expression vector (pMA6), and *glnR* was cloned into the pET28a expression vector (pRD153). The resulting pMA6 plasmid was transformed into *E*. *coli* Arctic Express cells, whereas pRD153 was introduced into *E*. *coli* BL21 pLysS cells.

The biomass of *E*. *coli* Arctic Express cells overexpressing MBP-NnaR was cultured in LB broth supplemented with 0.2% glucose, 100 µg/mL ampicillin and 0.01% gentamycin with the shaking at 37 °C until the OD_600_ reached 0.6, and the cells were cooled down. Expression of protein was induced by addition of 0.4 mM IPTG followed by overnight incubation at 6 °C. Cells were harvested and suspended in column buffer (20 mM Tris-HCl, 200 mM NaCl, 1 mM EDTA and 1 mM DTT) with 100 µg/mL lysozyme and 1 mM phenylmethylsulphonyl fluoride. For cell disruption, the suspension was sonicated, and the cell lysate was pre-cleared by centrifugation. Recombinant NnaR was obtained using affinity chromatography by passing the cell lysate through amylose resin (Amylose Resin High Flow), where the recombinant protein was washed with the column buffer and eluted with eluting buffer (20 mM Tris-HCl, 200 mM NaCl, 1 mM EDTA, 1 mM DTT and 10 mM maltose). Next, NnaR was purified on a HiTrap SP FF column using AKTA start (GE Healthcare) and was eluted in a NaCl gradient with buffers containing 50 mM Tris-HCl and 10% glycerol.

The *E*. *coli* BL21 pLysS cells overexpressing His-GlnR were cultured in LB broth supplemented with 0.005% kanamycin and 34 µg/mL chloramphenicol with shaking at 37 °C until the OD_600_ reached 0.6, and the cells were cooled down to 20 °C. Expression of protein was induced by adding 1 mM IPTG followed by incubation overnight at 20 °C. Cells were harvested, centrifuged and suspended in column buffer (50 mM Tris-HCl, 1 M NaCl, 0.1% Triton X-100, 10 mM imidazole and 10% glycerol; pH = 7.8) with 1 mM phenylmethylsulphonyl fluoride. Cell lysate was prepared as described above. His-tagged GlnR was obtained by applying affinity chromatography purification using HisPur Ni-NTA Resin (Thermo Fisher Scientific), where the recombinant protein was washed with the column buffer and eluted with eluting buffer (50 mM Tris-HCl, 500 mM NaCl, 0.5 M imidazole and 10% glycerol; pH = 7.8). Next, to remove imidazole, GlnR was passed through a Sephadex G-25 M column (GE Healthcare), after which it was eluted with the buffer containing 50 mM Tris-HCl, 500 mM NaCl and 10% glycerol.

### Pull-down assay

A pull-down assay was performed to investigate the interaction between NnaR and GlnR. The recombinant MBP-NnaR and His-GlnR were used to perform the experiment. BSA (albumin from bovine serum, SIGMA) was the control. Equimolar amounts of proteins (5 nM) were tested for interactions. In the first step, His-GlnR in the washing buffer (50 mM Tris, 150 mM NaCl, 10% glycerol, 0.1% Triton X-100, pH = 8) was bound to HisPur Ni-NTA magnetic beads (rotating for 1 hour at 4 °C). The protein-coated beads were collected on a magnetic separator, washed twice with the washing buffer and incubated with MBP-NnaR or BSA in the washing buffer (rotating for 2 hours at 4 °C). Next, the beads were collected on a magnetic separator and washed six times. The protein complexes were eluted in buffer containing 500 mM imidazole (50 mM Tris, 500 mM NaCl, 10% glycerol, pH = 8). Finally, the load fraction (5 µg of each protein), the last wash after incubation with MBP-NnaR or BSA and the eluted protein complexes were resolved by 12% SDS-PAGE and stained by Instant Blue to visualize the results.

### Phenotypic microarrays

To understand the metabolic differences between the studied strains, the BIOLOG Phenotype Microarray screening platform was applied as described previously^33^ with modifications. The *M*. *smegmatis* Δ*nnaR*, Δ*glnR*, and wild-type strains were grown in 7H9/OADC medium up to logarithmic phase. The cells were collected by centrifugation, washed three times in Sauton’s medium devoid of nitrogen sources, diluted to OD_600_ 0.2 (50 mL) and incubated with shaking agitation at 37 °C for 16 hours. Afterwards, the cells were collected again and used for preparing the suspension of bacterial cells according to the BIOLOG protocol. Every well of the PM 3-8 plates, which contained a total of 575 different growth conditions, was inoculated by adding 100 μL of the suspension of bacterial cells. Next, the PM plates were incubated at 30 °C in a BIOLOG Omnilog incubator for collecting raw kinetic values every 15 minutes for 72 hours. The area under the curve (AUC) values were collected form each well from two independent experiments. The obtained data were analysed using Microsoft Excel.

### Growth assays under variable nitrogen sources

*M*. *smegmatis* strains Δ*nnaR*, Δ*glnR*, Δ*nnaR-attB*::_*p*hsp60_*nnaR*, Δ*glnR-attB*::_*p*hsp60_*nnaR*, Δ(*nnaR, glnR*) and wild-type were grown in 7H9 medium supplemented with Tween 80 and OADC up to the logarithmic stage of growth. To induce nitrogen starvation, cells were washed twice in nitrogen-free Sauton medium. The OD_600_was adjusted to 0.2 in the same medium, and the cells were grown for 16 hours at 37 °C. Next, cells were harvested by centrifugation and diluted to OD_600_ 0.1 in Sauton’s medium supplemented with various substances that were tested as sole nitrogen sources: sodium nitrite (5 mM), sodium nitrate, acetamide (5 mM), ammonium sulphate, urea (pH 4.5), uric acid, histidine (pH 9.5), leucine (pH 9.5), allantoin, hydantoin, proline (pH 9.5), methionine (pH 9.5), L-glutamic acid (all at 10 mM concentration). 7H9 medium containing OADC and Tween 80 was used as a positive control, and nitrogen-free Sauton’s medium was the negative control. The kinetics of the growth was monitored by measuring absorbance at 600 nm at 3, 6, 9, 24, 36, 48 and 60 hours of growth. Cell viability was determined by measuring the colony-forming units (CFU) on 7H10 agar plates at the 24 hours timepoint. Plates were incubated at 37 °C for 3–5 days, colonies were counted, and data were plotted in Excel.

### Griess-Ilosvay assay

The ability to reduce nitrate to nitrites by Δ*nnaR*, Δ*glnR*, Δ*nnaR-attB*::_*p*hsp60_*nnaR*, Δ*glnR-attB*::_*p*hsp60_*nnaR* mutant strains and wild-type was evaluated using the Griess Reagent System (Promega). The samples were collected at 0, 6, 12, 24, 36, 48, 60 and 72 hours of growth on Sauton’s medium containing sodium nitrate as a sole nitrogen source. The level of nitrite in supernatants was measured in 96-well plates. The nitrite standard reference curve (100–1.56 µM) was prepared by two-fold serial dilutions of the 100 µM nitrite standard in nitrogen-free Sauton’s medium. Sulphanilamide solution (1% sulphanilamide in 5% phosphoric acid) was dispensed to experimental samples and nitrite standards, and plates were incubated in the dark for 10 minutes at room temperature. Next, the 0.1% N-1-napthylethylenediamine dihydrochloride (NED) solution (in water) was added to all wells, and the plates were incubated as above. The absorbance was measured at 550 nm by Benchmark Plus Microplate Spectrophotometer (BioRad). The nitrite standard reference curve was generated using Excel, and concentrations of nitrites were determined by comparing the average absorbance to the nitrite standard reference curve.

### RNA isolation, removal of rRNA and library preparation

*M*. *smegmatis* strains lacking *nnaR* or *glnR*, grown overnight in 7H9 liquid broth supplemented with OADC and Tween 80, were washed three times in nitrogen-free Sauton’s medium. Bacteria were diluted to an OD_600_ of 0.1 in the same medium supplemented with 1 mM (nitrogen-limiting) or 30 mM (nitrogen-excess) ammonium sulphate. Growth kinetics were monitored by measuring the OD_600_. To confirm nitrogen depletion in liquid cultures of Δ*nnaR*, Δ*glnR* and wild-type strains, the presence of ammonium ions was monitored during bacterial growth by the ammonium test (Merck) following the manufacturer’s protocol. Six millilitres of each culture was collected at the indicated time points and centrifuged, and the culture supernatant was used for testing the presence of ammonium ions in the growth media. Based on each sample’s colour compared to the blank on the colour card attached to the kit, the presence or absence of ammonium ions was determined. After the ammonium ions were depleted, cultures (50 mL) were centrifuged (4500 rpm for 10 min at room temperature), and the total RNA was isolated as described previously^33,40^. Briefly, Trizol LS reagent (Invitrogen) was used to extract RNA. Cells were disrupted twice using the MP disruptor system with the Quick prep adapter (MP Biomedicals) and 0.1 mm silica spheres (45 seconds, 6.0 m/s with 5 min intervals). DNase I turbo (Invitrogen by Thermo Fisher Scientific) was used to remove DNA contamination according to the manufacturer’s instructions. The RNA quantity was assessed using a NanoDrop 2000 spectrophotometer (Thermo Fisher Scientific), and RNA integrity was verified using an Agilent 2100 BioAnalyzer following the manufacturer’s protocol (Agilent RNA 6000 Nano Kit). Before rRNA removal, the RNA samples were purified using AMPure magnetic beads (Becton Dickinson). To remove rRNA from the samples, the Ribo-Zero rRNA Removal Kit (Illumina) was applied, and the libraries were prepared using the KAPA Stranded RNA-Seq kit (KAPA Biosystems) following the detailed method provided by the manufacturer. The resulting libraries were examined on an Agilent 2100 BioAnalyzer on a DNA 1000 chip and later quantified by qPCR with the NEBNext® Library Quant Kit for Illumina (New England Biolabs). The libraries were sequenced using the NextSeq500 System from Illumina with the NextSeq. 500/550 Mid Output v2 sequencing kit (150 cycles, Illumina) ensuring around 5 mln pair-end reads per sample. Experiments were performed in triplicate, averaged results are shown.

### Bioinformatics

The bioinformatic analyses of the total RNA sequencing results were performed in-house using an array of scripts and programmes. The sequencing indexes were initially removed using Cutadapt software^41^, and quality trimming of sequencing reads was performed with help of the Sickle programme^42^. Trimmed reads were next mapped to the *M*. *smegmatis* genome (NC_008596.1, from NCBI; source: https://www.ncbi.nlm.nih.gov/nuccore/NC_008596) using the Bowtie2 short sequence aligner^43^. Mapped reads derived from the bacterial RNA were counted to the corresponding genes using HTSeq-count script^44^. The differences between the tested conditions were examined using the online Degust RNA-Seq analysis platform with default parameters (http://degust.erc.monash.edu/, originally designed by D. R. Powell). Statistical analysis of differential gene expression (DGE) was performed using the empirical Bayes quasi-likelihood F-test. For all DGE analysis carried out in this study, genes having a false discovery rate (FDR) of <0.05 and a log2 fold change of >1.5 were considered significantly differential.

### Quantitative real-time (qRT) PCR

The qRT-PCR technique was applied as a validation experiment of RNA-Seq data. The transcript levels for selected genes were studied using the Maxima SYBR green qPCR master mix (Thermo Fisher Scientific) and 7900HT real time PCR system (Applied Biosystems). The reverse transcription reaction and qRT-PCR were carried out as described previously^20,33^. Briefly, the SuperScript III First-Strand Synthesis Super Mix kit with random hexamers (Invitrogen) was used for reverse transcription of 1 µg of total RNA according to the manufacturer’s protocol. Each qPCR reaction (total volume of 25 µl) contained: 1 x Maxima SYBR green qPCR master mix, 0.3 μM each primer, 50 ng of cDNA (primers sequences used for qRT-PCR are listed in Table S1). Real-time PCR conditions were as follows: initial activation at 95 °C for 10 min, followed by 40 cycles at 95 °C for 15 sec (denaturation), 62 °C for 30 sec (annealing), 72 °C for 30 sec (extension). The melting curve analysis was performed at the end of each qPCR reaction to verify a single, specific product was generated. The threshold cycle (C_T_) value for each studied gene was normalized to the expression of *msmeg*_2758 (*sigA*) (ΔC_T_) and converted to linear form (2^−ΔCT^). The RNA samples for each strain were isolated from three independently grown cultures.

### Electrophoretic mobility shift assay (EMSA)

Interactions of MBP-NnaR and His-GlnR with the hexachlorofluorescein-labelled promoter regions of *msmeg_0427*, *msmeg_0433*, *msmeg_4008*, *msmeg_5360*, *msmeg_5765* were assessed using EMSA. First, 0 µM NnaR, 2 µM NnaR, 4 µM GlnR or 2 µM NnaR combined with 4 µM GlnR was incubated with 30 nM of labelled DNA in buffer containing 10 mM Tris, 1 mM DTT, 2.5% glycerol, 20 mM MgCl_2_, 500 ng poly(dI·dC), and 0.05% NP-40 (pH 7.5). Samples were incubated for 10 min at room temperature and resolved on 2% agarose gels. The protein-DNA complexes were visualized by a GE Typhoon 8600 Imager Scanner.

To confirm the specificity of the shifts, competitive EMSA for the promoter region of *msmeg_0427* was performed. To do this, 30 nM of the labelled region of *msmeg_0427* and the mixture of labelled (30 nM) with 100-fold excess of unlabelled specific DNA was incubated with 2 µM of MBP-NnaR for 10 min at room temperature. The complexes were resolved and visualized as above.

### Biofilm formation

*M*. *smegmatis* strains were cultured in Middlebrook 7H9 (Difco Laboratories) liquid media supplemented with 10% OADC, 0.05% Tween 80 and kanamycin (25 µg/mL) when necessary. Pellicle biofilms of tested here strains were grown as described earlier^22,23^. 10 µls of a saturated planktonic culture of each strain was inoculated into 10 mL of detergent-free Sauton’s_rich_, Sauton’s_N1/2_ and Sauton’s_N0_ media and transferred onto 6-well polystyrene plates. The biofilms were incubated at 30 °C for up to 7 days.

### Statistical analysis

Statistical analyses in this study were carried out with GraphPad Software (La Jolla, CA, USA) for Windows.

## Electronic supplementary material

Supplementary Materials

Supplementary Table S2

Supplementary Table S3

## References

[1] Parise-Fortes Maria, Lastória Joel, Marques Silvio, Putinatti Maria Stella, Stolf Hamilton, Marques Mariângela Ester, Haddad Vidal (2014). Lepromatous leprosy and perianal tuberculosis: a case report and literature review. Journal of Venomous Animals and Toxins including Tropical Diseases.

[2] Amon J, Titgemeyer F, Burkovski A (2009). A Genomic View on Nitrogen Metabolism and Nitrogen Control in Mycobacteria. J Mol Microb Biotech.

[3] Jenkins Victoria A, Barton Geraint R, Robertson Brian D, Williams Kerstin J (2013). Genome wide analysis of the complete GlnR nitrogen-response regulon in Mycobacterium smegmatis. BMC Genomics.

[4] Williams Kerstin J, Bryant William A, Jenkins Victoria A, Barton Geraint R, Witney Adam A, Pinney John W, Robertson Brian D (2013). Deciphering the response of Mycobacterium smegmatis to nitrogen stress using bipartite active modules. BMC Genomics.

[5] Williams KJ (2015). Deciphering the metabolic response of Mycobacterium tuberculosis to nitrogen stress. Mol Microbiol.

[6] Beckers G (2005). Regulation of AmtR-controlled gene expression in Corynebacterium glutamicum: mechanism and characterization of the AmtR regulon. Mol Microbiol.

[7] Petridis, M., Benjak, A. & Cook, G. M. Defining the nitrogen regulated transcriptome of Mycobacterium smegmatis using continuous culture. *Bmc Genomics* **16**, 10.1186/S12864-015-2051-X (2015). 10.1186/s12864-015-2051-x PMC4617892 26482235

[8] Tiffert Y (2011). Proteomic analysis of the GlnR-mediated response to nitrogen limitation in Streptomyces coelicolor M145. Appl Microbiol Biot.

[9] Burkovski A (2007). Nitrogen control in Corynebacterium glutamicum: proteins, mechanisms, signals. J Microbiol Biotechnol.

[10] Amon J (2008). Nitrogen Control in Mycobacterium smegmatis: Nitrogen-Dependent Expression of Ammonium Transport and Assimilation Proteins Depends on the OmpR-Type Regulator GlnR. J Bacteriol.

[11] Malm S (2009). The roles of the nitrate reductase NarGHJI, the nitrite reductase NirBD and the response regulator GlnR in nitrate assimilation of Mycobacterium tuberculosis. Microbiol-Sgm.

[12] Liu, X. X., Shen, M. J., Liu, W. B. & Ye, B. C. GlnR-Mediated Regulation of Short-Chain Fatty Acid Assimilation in Mycobacterium smegmatis. *Front Microbiol* **9**, 10.3389/Fmicb.2018.01311 (2018). 10.3389/fmicb.2018.01311 PMC6023979 29988377

[13] Liu, X. -X., Liu, W. -B., Shen, M. -J. & Ye, B. -C. Nitrogen regulator GlnR directly controls transcription of prpDBC operon involved in methylcitrate cycle in Mycobacterium smegmatis. *bioRxiv*, 10.1101/353219 (2018). 10.1128/JB.00099-19 PMC6436344 30745367

[14] Amin R, Reuther J, Bera A, Wohlleben W, Mast Y (2012). A novel GlnR target gene, nnaR, is involved in nitrate/nitrite assimilation in Streptomyces coelicolor. Microbiology.

[15] Bretl DJ, Demetriadou C, Zahrt TC (2011). Adaptation to environmental stimuli within the host: two-component signal transduction systems of Mycobacterium tuberculosis. Microbiol Mol Biol Rev.

[16] Jenkins VA, Robertson BD, Williams KJ (2012). Aspartate D48 is essential for the GlnR-mediated transcriptional response to nitrogen limitation in Mycobacterium smegmatis. FEMS Microbiol Lett.

[17] Amin R (2016). Post-translational Serine/Threonine Phosphorylation and Lysine Acetylation: A Novel Regulatory Aspect of the Global Nitrogen Response Regulator GlnR in S. coelicolor M145. Front Mol Biosci.

[18] Sassetti CM, Boyd DH, Rubin EJ (2003). Genes required for mycobacterial growth defined by high density mutagenesis. Molecular Microbiology.

[19] Parish T, Stoker N (2000). G. glnE is an essential gene in Mycobacterium tuberculosis. J Bacteriol.

[20] Pawelczyk, J., Viljoen, A., Kremer, L. & Dziadek, J. The influence of AccD5 on AccD6 carboxyltransferase essentiality in pathogenic and non-pathogenic Mycobacterium. *Sci Rep-Uk* **7**, 10.1038/Srep42692 (2017). 10.1038/srep42692 PMC5311964 28205597

[21] Hetherington SV, Watson AS, Patrick CC (1995). Sequence and analysis of the rpoB gene of Mycobacterium smegmatis. Antimicrob Agents Chemother.

[22] Yang Y (2017). Defining a temporal order of genetic requirements for development of mycobacterial biofilms. Molecular Microbiology.

[23] Yang, Y., Richards, J. P., Gundrum, J. & Ojha, A. K. GlnR Activation Induces Peroxide Resistance in Mycobacterial Biofilms. *Front Microbiol* **9**, 10.3389/Fmicb.2018.01428 (2018). 10.3389/fmicb.2018.01428 PMC6039565 30022971

[24] Sola-Landa A, Rodriguez-Garcia A, Amin R, Wohlleben W, Martin JF (2013). Competition between the GlnR and PhoP regulators for the glnA and amtB promoters in Streptomyces coelicolor. Nucleic Acids Res.

[25] Yao LL (2014). GlnR-mediated regulation of nitrogen metabolism in the actinomycete Saccharopolyspora erythraea. Appl Microbiol Biot.

[26] Rajagopalan M (2010). Mycobacterium tuberculosis origin of replication and the promoter for immunodominant secreted antigen 85B are the targets of MtrA, the essential response regulator. The Journal of biological chemistry.

[27] Gorla, P. *et al*. MtrA response regulator controls cell division and cell wall metabolism and affects susceptibility of mycobacteria to the first line antituberculosis drugs. *Front Microbiol* **9**, 10.3389/fimcb.2018.02839 (2018). 10.3389/fmicb.2018.02839 PMC6265350 30532747

[28] Minch, K. J. *et al*. The DNA-binding network of Mycobacterium tuberculosis. *Nature communications* **6**, 10.1038/Ncomms6829 (2015). 10.1038/ncomms6829 PMC4301838 25581030

[29] Wang Y (2013). Three of Four GlnR Binding Sites Are Essential for GlnR-Mediated Activation of Transcription of the Amycolatopsis mediterranei nas Operon. J Bacteriol.

[30] Yao LL, Ye BC (2016). Reciprocal Regulation of GlnR and PhoP in Response to Nitrogen and Phosphate Limitations in Saccharopolyspora erythraea. Appl Environ Microb.

[31] JeSsberger N (2013). Nitrogen starvation-induced transcriptome alterations and influence of transcription regulator mutants in Mycobacterium smegmatis. BMC Res Notes.

[32] Xu Y, You D, Ye B-C (2017). Nitrogen regulator GlnR directly controls transcription of genes encoding lysine deacetylases in Actinobacteria. Microbiology.

[33] Dadura K (2017). PdtaS Deficiency Affects Resistance of Mycobacteria to Ribosome Targeting Antibiotics. Front Microbiol.

[34] Savard P-Y (2011). Structure and dynamics of Mycobacterium tuberculosis truncated hemoglobin N: insights from NMR spectroscopy and molecular dynamics simulations. Biochemistry.

[35] Ouellet H (2002). Truncated hemoglobin HbN protects Mycobacterium bovis from nitric oxide. Proc Natl Acad Sci USA.

[36] Pawaria S, Lama A, Raje M, Dikshit KL (2008). Responses of Mycobacterium tuberculosis hemoglobin promoters to *in vitro* and *in vivo* growth conditions. Appl Environ Microb.

[37] Joseph SV, Madhavilatha GK, Kumar RA, Mundayoor S (2012). Comparative analysis of mycobacterial truncated hemoglobin promoters and the groEL2 promoter in free-living and intracellular mycobacteria. Appl Environ Microb.

[38] Sambrook, J. *Molecular cloning: a laboratory manual/Joseph Sambrook*, *David W*. *Russell*. (Cold Spring Harbor Laboratory, 2001).

[39] Plocinska R (2012). Septal Localization of the Mycobacterium tuberculosis MtrB Sensor Kinase Promotes MtrA Regulon Expression. The Journal of biological chemistry.

[40] Pawelczyk J (2011). AccD6, a key carboxyltransferase essential for mycolic acid synthesis in Mycobacterium tuberculosis, is dispensable in a nonpathogenic strain. J Bacteriol.

[41] Martin Marcel (2011). Cutadapt removes adapter sequences from high-throughput sequencing reads. EMBnet.journal.

[42] Joshi, N. A. & Fass, J. N. Sickle: A sliding-window, adaptive, quality-based trimming tool for FastQ files (Version 1.33) [Software]. Available at, https://github.com/najoshi/sickle (2011).

[43] Langmead B, Salzberg SL (2012). Fast gapped-read alignment with Bowtie 2. Nature methods.

[44] Anders S, Pyl PT, Huber W (2015). HTSeq–a Python framework to work with high-throughput sequencing data. Bioinformatics.

