# Supplementary material for: The NnaR orphan response regulator is essential for the utilization of nitrate and nitrite as sole nitrogen sources in mycobacteria
Source: Sci Rep. 2018 Dec 3;8:17552. doi: 10.1038/s41598-018-35844-z (PMC6277429; doi:10.1038/s41598-018-35844-z)

**Supplementary Data:**

**The NnaR orphan response regulator is essential for the utilization of nitrate and nitrite as sole nitrogen sources in mycobacteria**

Magdalena Antczak^1^§, Renata Płocińska^1^§, Przemysław Płociński^1^, Anna Rumijowska-Galewicz^1^,Anna Żaczek^2^, Dominik Strapagiel^3^, Jarosław Dziadek^1^

1. **Figures and Legends**
2. **Tables**
3. **The uncropped versions of blots used in this study**


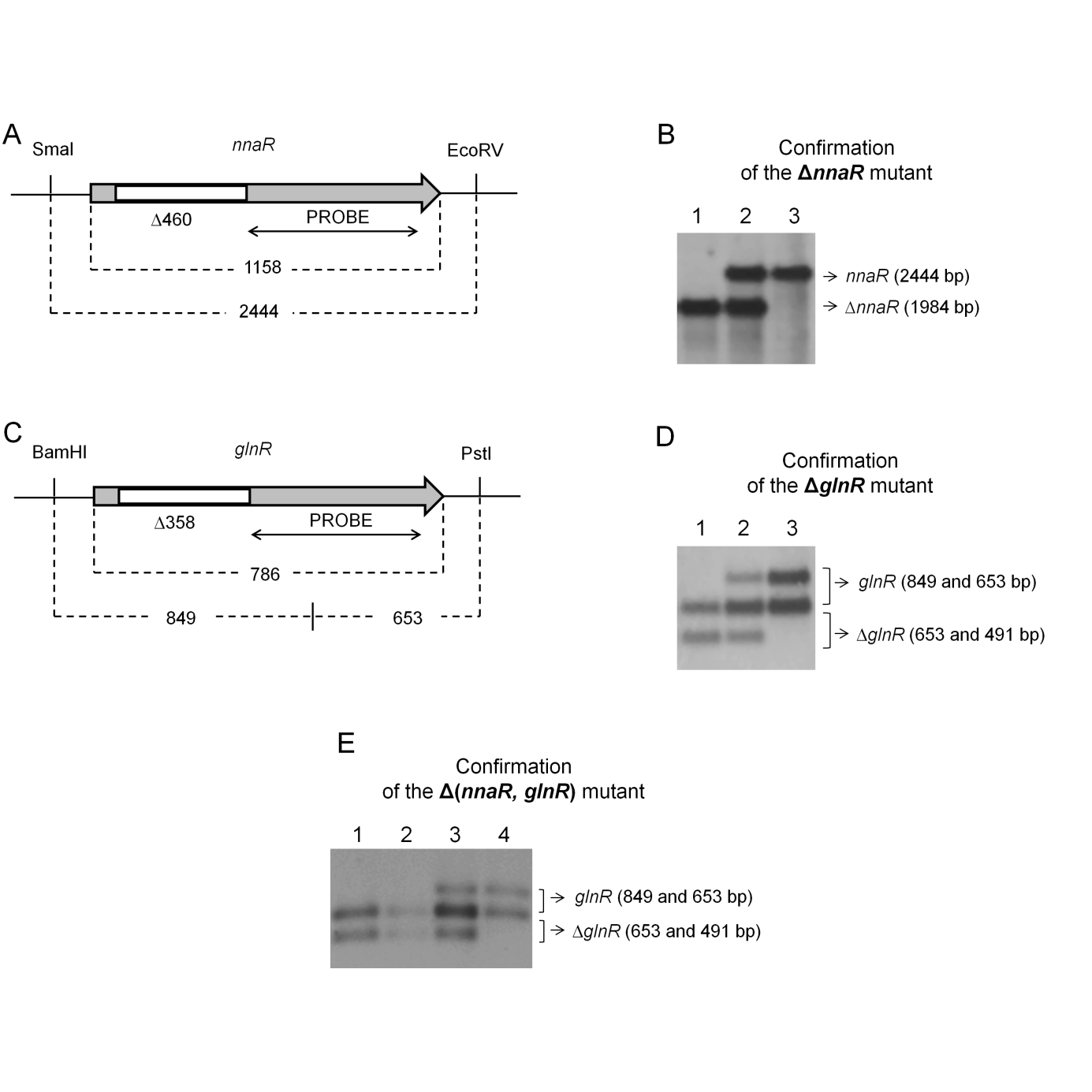


**Fig. S1.** Confirmation of mutant construction by Southern Blotting analysis. Fragments of genomic DNA containing (**A**) the *nnaR* (1158 bp) and (**C**) *glnR* (786 bp) genes are shown. Restriction sites and internal deletion fragments are indicated. The *nnaR* and *glnR* genes were presented as grey arrows with white blocks constituting deletions (460 and 358 bp, respectively). Southern Blot autoradiograms show the presence of wild *nnaR* (2444 bp) (**B; lane 3**) and *glnR* (849 and 653 bp) (**D; lane 3** and **E; 4**) copies in a wild type *M. smegmatis*, mutated *nnaR* (1984 bp) (**B; 1**) and *glnR* (653 and 491 bp) (**D; 1** and **E; 1, 2**) in double crossover mutants and both wild type and mutated copies of *nnaR* (2444 and 1984 bp) (**B; 2**) and *glnR* (849, 653 and 491 bp) (**D; 2** and **E;3**) in single crossover strains.


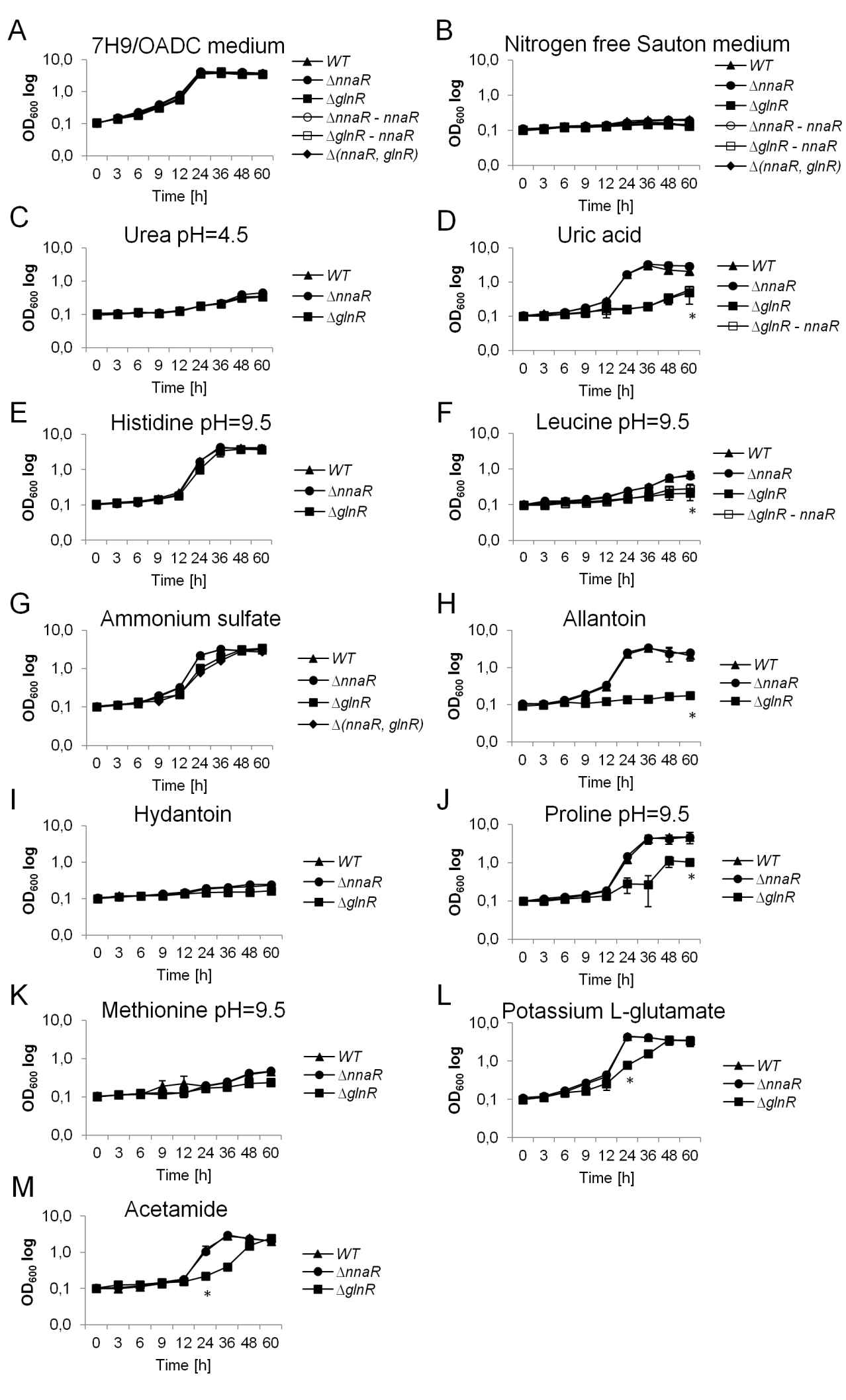


**Fig. S2.** Kinetics of growth of mutant strains and wild type *M. smegmatis* propagated on nitrogen-limiting Sauton medium containing various nitrogen sources. Wild type, ∆*nnaR*, ∆*glnR,* ∆*nnaR–attB::_phsp_nnaR* (∆*nnaR-nar*)*,* ∆*glnR-attB::_phsp_nnaR* (∆*glnR-nar*) and ∆(*nnaR*, *glnR*) were grown in the presence of the (**C**) urea (pH=4.5), (**D**) uric acid, (**E**) histidine (pH=9.5), (**F**) leucine, (**G**) ammonium sulfate, (**H**) allantoin, (**I**) hydantoin, (**J**) proline, (**K**) methionine, (**L**) L-glutamic acid potassium salt monohydrate and (**M**) acetamide (5mM) at 10 mM final concentration. Standard (**A**) 7H9/OADC medium was used as a positive control and (**B**) nitrogen free Sauton medium as a negative control. Growth of strains was determined by measuring the OD_600_ at indicated time points and was shown on the graphs as averages from three independent experiments ± standard deviation. The statistical significance was determined using Student’s *t*-test (* *p* < 0.03): (**D)** for ∆*glnR*, ∆*glnR– attB::_phsp_nnaR* *p* < 0.001, (**F)** for ∆*glnR p* = 0.003, ∆*glnR– attB::_phsp_nnaR p* = 0.002, (**H**) for ∆*glnR* *p* = 0.005, (**J**) for ∆*glnR p* = 0.03, (**L**) for ∆*glnR p* = 0.008, (**M**) for ∆*glnR p* < 0.005.


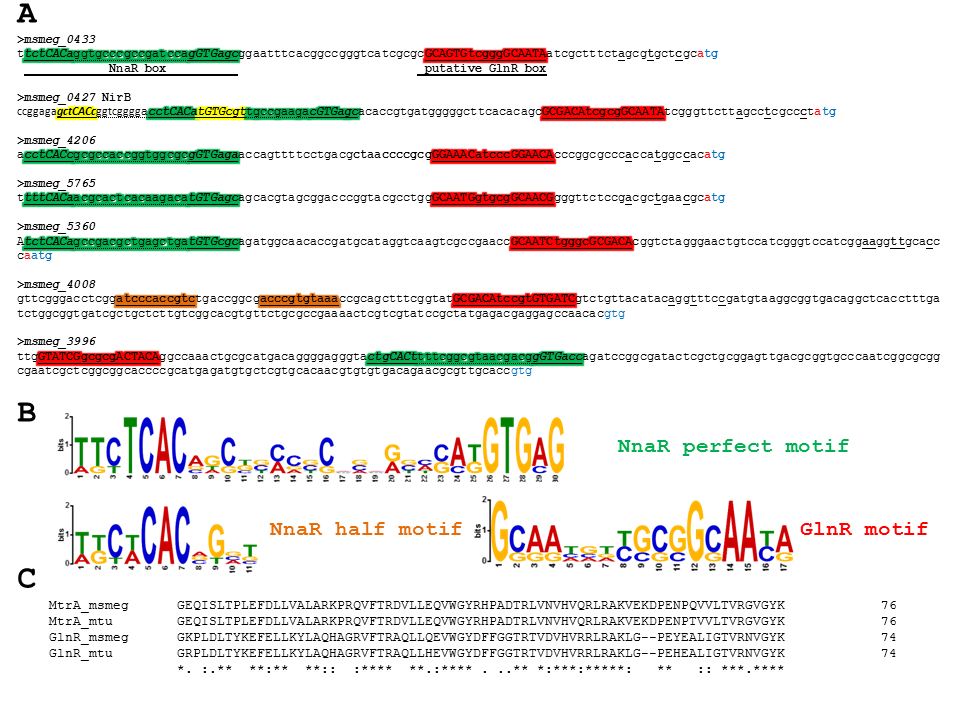


**Fig. S3.** MEME prediction of DNA-binding motifs for NnaR- and GlnR-dependent regulation of gene expression based on RNA-Seq results. (**A**) The putative promoter sequences for the annotated gene, or the first gene in the operon, were analysed. Fractions of the sequences containing the predicted regulatory motifs are shown. Targets were chosen based on the comparison of the Δ*nnaR* and wild-type transcriptomic profiles obtained upon nitrogen depletion. The start codons are marked with blue font and the predicted transcription start sites are represented by single red letters. (**B**) Separate MEME analysis was performed for the set of transcripts downregulated by more than a log2 fold value of -1.5 in the RNA-Seq experiment (predicted half-motif for NnaR binding) and for the subset of genes downregulated by more than a log2 fold value of ±2 (predicted perfect motif for NnaR and the NnaR-associated GlnR-binding motif). (**C**) Clustal omega alignment of DNA-binding domains of GlnR and MtrA from *M. smegmatis* and *M. tuberculosis*.


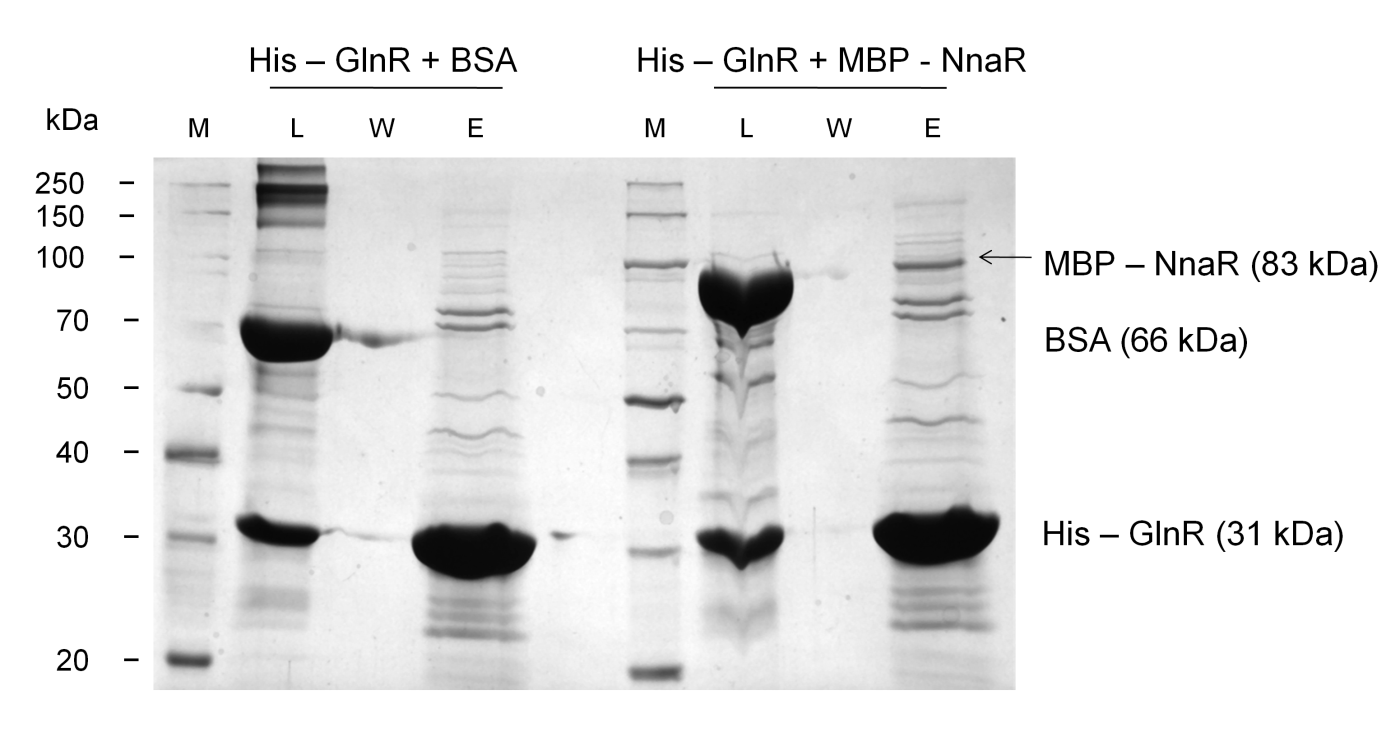


**Fig. S4.** Interaction of GlnR-BSA and GlnR-NnaR analyzed by pull-down assay on HisPur Ni-NTA magnetic beads. Equimolar amounts of proteins were used. Protein ladder (M), load (L), wash (W) and elution fractions (E) were resolved on a 12 % SDS-PAGE gel and stained with Instant Blue.


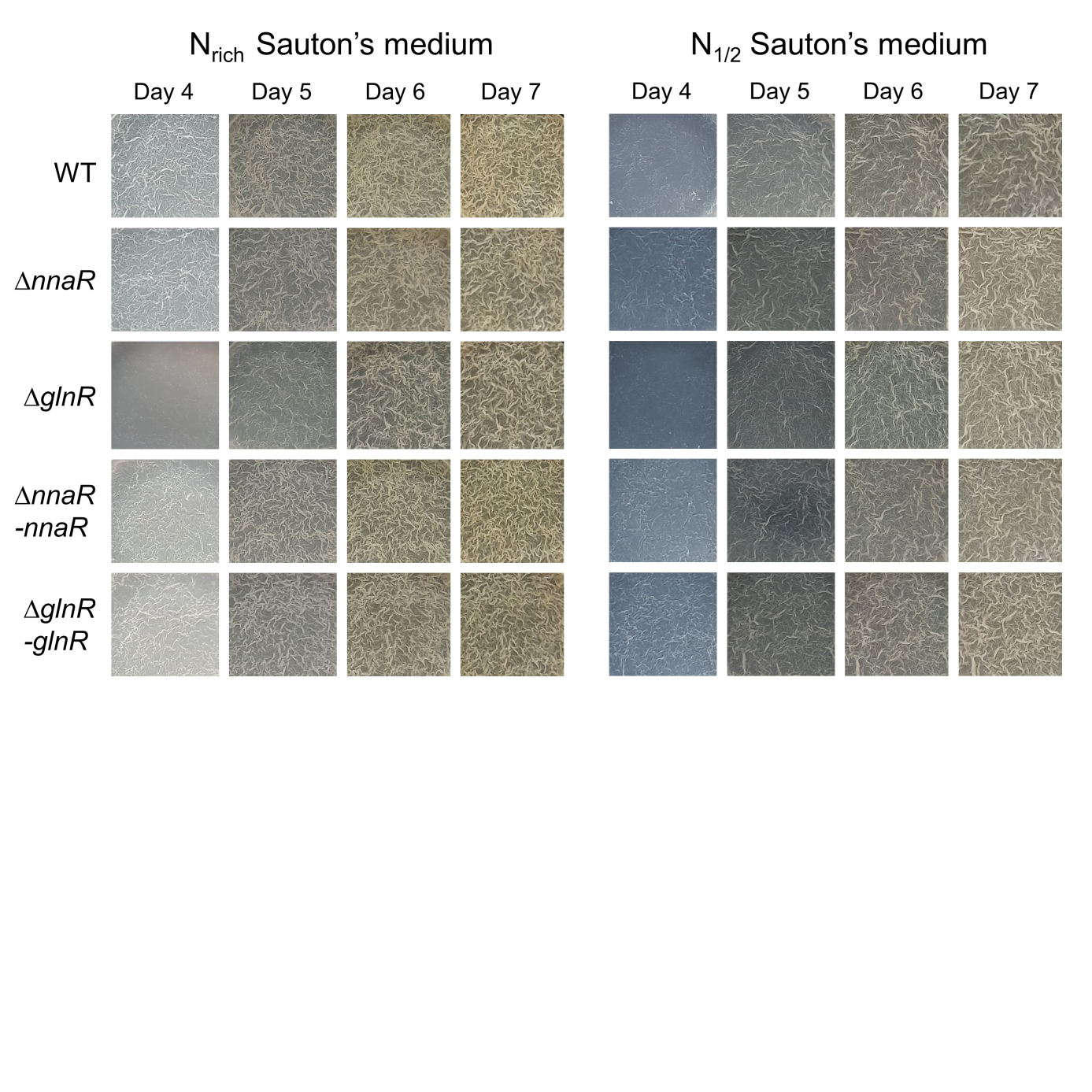


**Fig. S5.** Biofilms of the wild type, Δ*nnaR*, Δ*glnR* and complemented strains in N_rich_ and N_1/2_ versions of Sauton’s medium at the indicated time points.

**Table S1**

| **A. Primers used in this study** |  |  |
| --- | --- | --- |
| Name of primer | Sequence (5’>3’) | Application |
|  | **Primers used to amplify DNA for targeted gene replacement** |  |
| Msmeg_0432GR1ScaI-F | CAGTACTCAGTGTCGGGGCAATAATCGC |  |
| Msmeg_0432GR2HindIII-R | CAAGCTTCGCACAGTTCATCGGCTCGT | *nnaR* knock-out |
| Msmeg_0432GR3HindIII-F | CAAGCTTGTCGCGGGTGCAGAGGTG |  |
| Msmeg_0432GR4KpnI-R | CGGTACCGTTCACCGCGCCGTTTGC |  |
| Msmeg_0432-probe-F | CAGATCTATGCGTGAGCCCGACTGGGC | Southern blotting - hybridization probe |
| Msmeg_0432-probe-R | CTCTAGACTCATAACGCGCGTGCCATCTCTG |  |
| Msmeg_5784GR1KpnI-F | TGGGGACGCCGCTGGTGC |  |
| Msmeg_5784GR2HindIII-R | CAAGCTTGCTGTCCGGACCGTGTGGGC | *glnR* knock-out |
| Msmeg_5784GR3HindIII-F | CAAGCTTCTCACCTACAAGGAATTCGAGCTCC |  |
| Msmeg_5784GR4ScaI-R | CAGTACTGGGGTTGTAGGAGAGGTTCTTGC |  |
| Msmeg_5784-probe-F | CGGATCCTTGGATCTACTGCTACTGACCGTCGACC | Southern blotting - hybridization probe |
| Msmeg_5784-probe-R | CTCTAGACTCACTGACTGGTCAACCGCCCC |  |
|  | **Primers used for clonings** |  |
| Msmeg_0432BglII-F | CAGATCTATGCGTGAGCCCGACTGGGC | *nnaR* complementation |
| Msmeg_0432HindIII-R | CAAGCTTCATAACGCGCGTGCCATCTCTG |  |
| Msmeg_5784NotI-F | AGCGCCGCGTCCTCGCGGATCAGCCACGAG | *glnR*  complementation |
| Msmeg_5784HindIII-R | AAAGCTTCACTGACTGGTCAACCGCCC |  |
| Msmeg_0432MAL-KpnI-F | CGGTACCGATGCGTGAGCCCGACTGGGC | NnaR  overproduction |
| Msmeg_0432MAL-HindIII-R | CAAGCTTCATAACGCGCGTGCCATCTCTG |  |
| Msmeg_5784pET-NdeI-F | ACATATGGATCTACTGCTACTGACCGT | GlnR  overproduction |
| Msmeg_5784pET-HindIII-R | AAGCTTCACTGACTGGTCAACCGCC |  |
|  | **Primers used for qRT_PCR** |  |
| Msmeg_0433qRT-F | TGATGACCGTCGTGTTGATG | *msmeg_0433* transcripts |
| Msmeg_0433qRT-R | GGGTAGAAGGCGTTGATGTT |  |
| Msmeg_0427qRT-F | CGCTACGGACAACAGGATT | *msmeg_0427* transcripts |
| Msmeg_0427qRT-R | AACCCGAAACACCCATCTT |  |
| Msmeg_5765qRT-F | AACATGTCACGTCTCAAGGG | *msmeg_5765* transcripts |
| Msmeg_5765qRT-R | CGAAGTGGTGCATCGTGATA |  |
| Msmeg_5360qRT-F | CGGTCATGCTGTTCTTCTACA | *msmeg_05360* transcripts |
| Msmeg_5360qRT-R | GATCTCGTTCCAGATGAAGTAGTC |  |
| Msmeg_1367qRT-F | GTCGACGAGTGCAAAGACAA | *msmeg_1367* transcripts |
| Msmeg_1367qRT-R | TGGTCTCGTCGAAGTACACG |  |
| Msmeg_1813qRT-F | GAAGTTGGTGCTGCGATGCTTGGC | *msmeg_1813*  transcripts |
| Msmeg_1813qRT-R | GGTGCACGCCAAAGGCAAACTCAC |  |
| Msmeg_2425qRT-F | TGGCGTTCTTCTACGGCGGAC | *msmeg_2425* transcripts |
| Msmeg_2425qRT-R | TGGCCGGACGAGAACGACATC |  |
| Msmeg_2982qRT-F | ACACGTCCGGCCCCACCATC | *msmeg_2982*  transcripts |
| Msmeg_2982qRT-R | CCGTCCTCGCCGATCAGTTCG |  |
| Msmeg_0432qRT-F | AAGGCTCGCATCGTGTCCAG | *msmeg_0432*  transcripts |
| Msmeg_0432qRT-R | AGGGAACGGATCCCAGTCGT |  |
| Msmeg_5784qRT-F | GCGGGAACGCCAATCAGGAA | *msmeg_5784*  transcripts |
| Msmeg_5784qRT-R | TGCTGCGCGAGGTACTTGAG |  |
|  | **Primers used for EMSA** |  |
| Msmeg_0433-F | [HEX]GACCCCTGGTGACCGCGTAAC | *msmeg_0433* promoter |
| Msmeg_0433-R | CATGCGAGCACGCTAGAAAGC |  |
| Msmeg_0427-F | [HEX]GGCGGTTGTGGCCTGACTGT | *msmeg_0427* promoter |
| Msmeg_0427-F | CGTGAGCACACCGTGATGGG |  |
| Msmeg_0427-R | CATAGGGCGAGGCTAAGAACCC |  |
| Msmeg_5765-F | [HEX]CGTGTGGCCCTTTCGTTCGATC | *msmeg_5765* promoter |
| Msmeg_5765-R | CATGCGTTCAGCGTCGGAGAAC |  |
| Msmeg_5360-F | [HEX]ATCTGCATCATCTCGCGACTTTG | *msmeg_05360* promoter |
| Msmeg_5360-R | CATTGGGTGCAACCTTCCGAT |  |
| Msmeg_4008-F | [HEX]GCAGCTTGGACCACCTATTT | *msmeg_4008*  promoter |
| Msmeg_4008-R | TCGATGGTGTCGACGAAGTA |  |
| **B. Strains constructed for this study** |  |  |
| Name | Description | Reference |
|  | **STRAINS** |  |
| Top10F’ | *Escherichia coli* strain | Invitrogen |
| Mc^2^155 | *M. smegmatis* wild type | Laboratory stock |
| BL21 (DE3) pLysS | *E. coli* strain | Novagen |
| Arctic Express | *E. coli* strain | Laboratory stock |
| Δ*nnaR* | *M. smegmatis nnaR* deletion strain | This study |
| ∆*nnaR*::*_p_*_hsp60_*nnaR* | *M. smegmatis nnaR* deletion strain carrying complementation plasmid | This study |
| Δ*glnR* | *M. smegmatis glnR* deletion strain | This study |
| Δ*glnR:: _p_*_glnR_*glnR* | *M. smegmatis glnR* deletion strain carrying complementation plasmid | This study |
| Δ*glnR:: _p_*_hsp60_*nnaR* | *M. smegmatis glnR* deletion strain carrying complementation plasmid | This study |
| Δ(*nnaR, glnR*) | *M. smegmatis nnaR* and *glnR* deletion strain | This study |
| **Plasmids used for this study** | **CLONING VECTORS** |  |
| pJET 1.2/blunt | Blunt cloning vector, AmpR | Thermo Scientific |
| p2NIL | Recombination vector, nonreplicating in mycobacteria, Kan^R^ | Parish& Stoker, 2000 |
| pGoal17 | Source of PacI cassette, Amp^R^ | Parish& Stoker, 2000 |
| pMV261 | Mycobacterial replicating vector carrying heat shock hsp60 promoter, Kan^R^ | Med-Immune Inc |
| pMV306K | Mycobacterial integrating vector, Kan^R^ | Med-Immune Inc |
| pMALC4e | *E. coli* expression vector carrying fusion with maltose binding protein, Amp^R^ | New England Biolabs |
| pET28a | *E. coli* expression vector carrying fusion with His-tag, Kan^R^ | Novagen |
| pMA1 | 1560 bp upstream fragment of *nnaR* gene cloned in p2Nil vector, Kan^R^ | This study |
| pMA2 | Δ *nnaR* and its flanking regions cloned in p2Nil vector, Kan^R^ | This study |
| pMA3 | pMA2 carrying PacI cassette, Kan^R^ | This study |
| pMA4 | *nnaR* cloned into pMV261vector, Kan^R^ | This study |
| pMA5 | *nnaR* with hsp60 promoter cloned into pMV306K vector, Kan^R^ | This study |
| PMA6 | *nnaR* cloned into pMALC4e vector | This study |
| pRD150 | 1484 bp upstream fragment of *glnR* gene cloned in p2Nil vector, Kan^R^ | This study |
| pRD151 | Δ *glnR* and its flanking regions cloned in p2Nil vector, Kan^R^ | This study |
| pRD152 | pRD151 carrying PacI cassette, Kan^R^ | This study |
| pRD153 | *glnR* cloned into pET28a vector | This study |
| pRD154 | *glnR* with glnR promoter cloned into pMV306 Kan^R^ vector | This study |

Uncropped versions of figure panels

Figure S1B


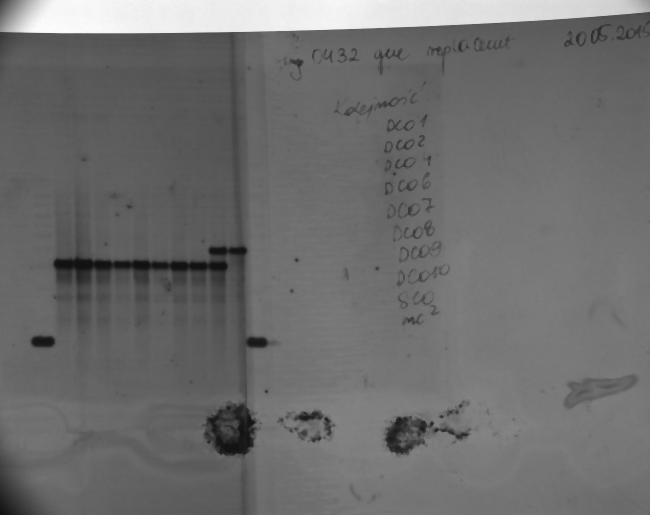


Figure S1D


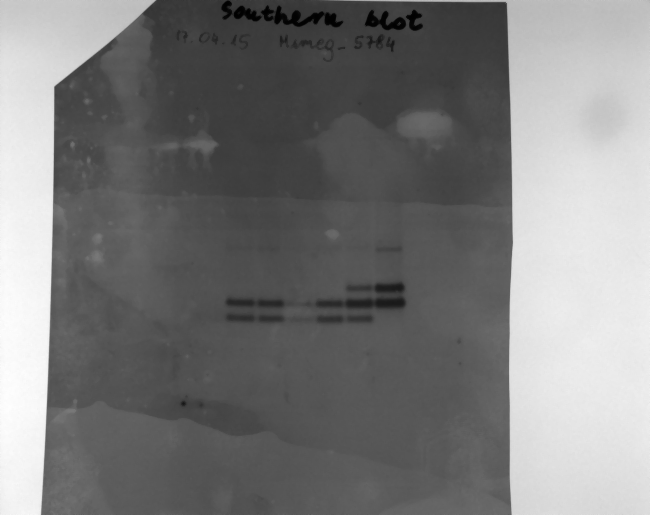


Figure S1E


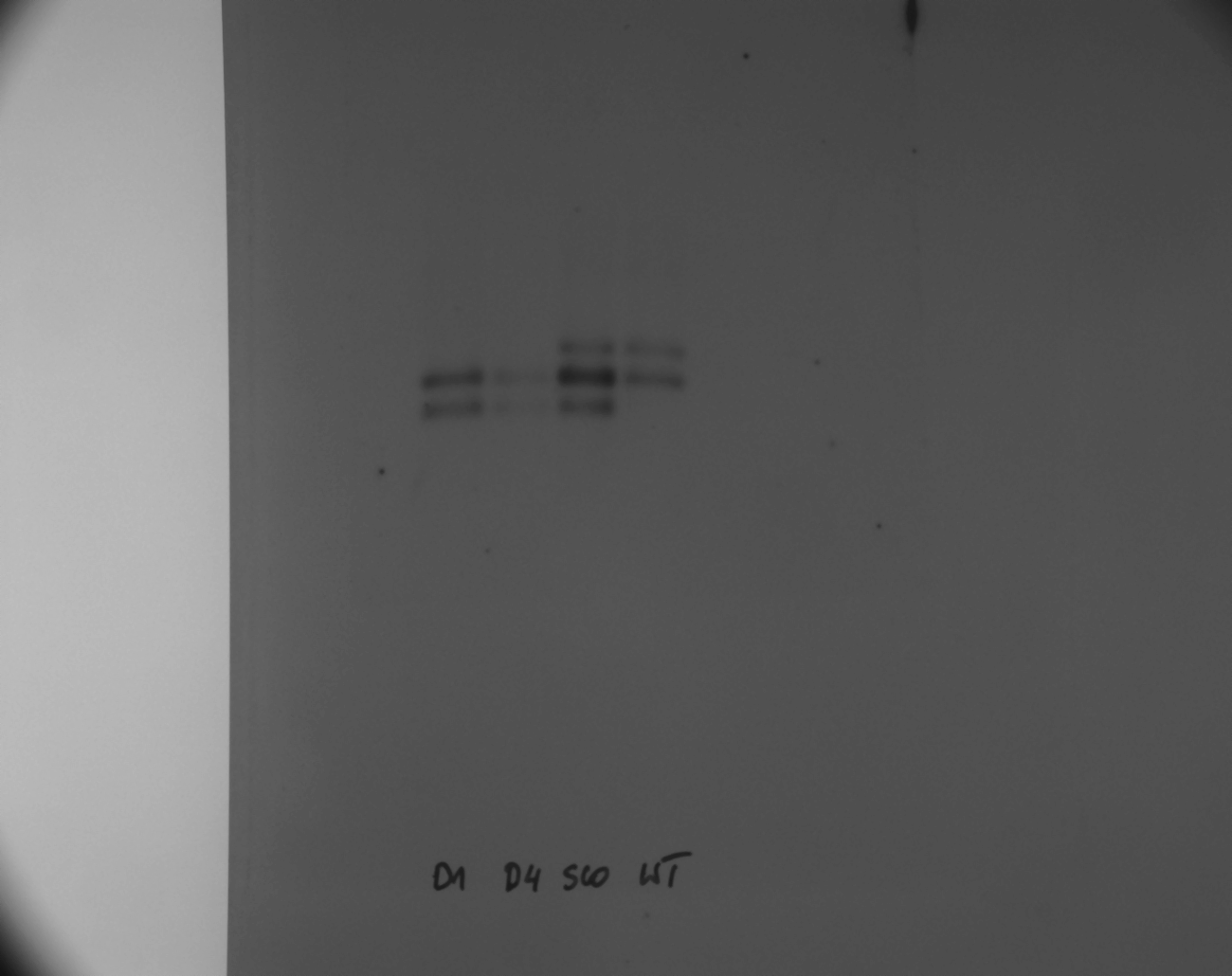


Figure 4A


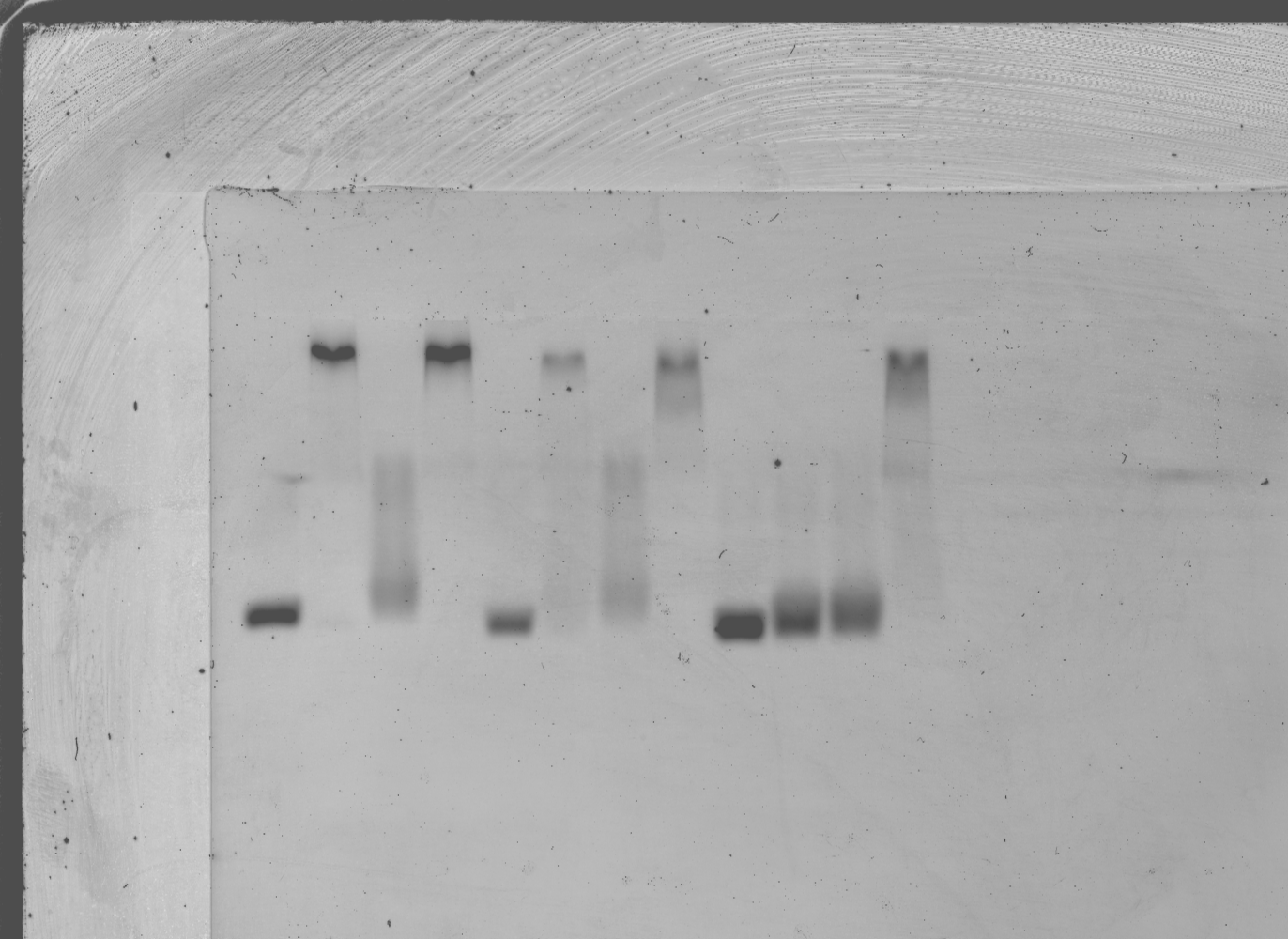


Figure 4A


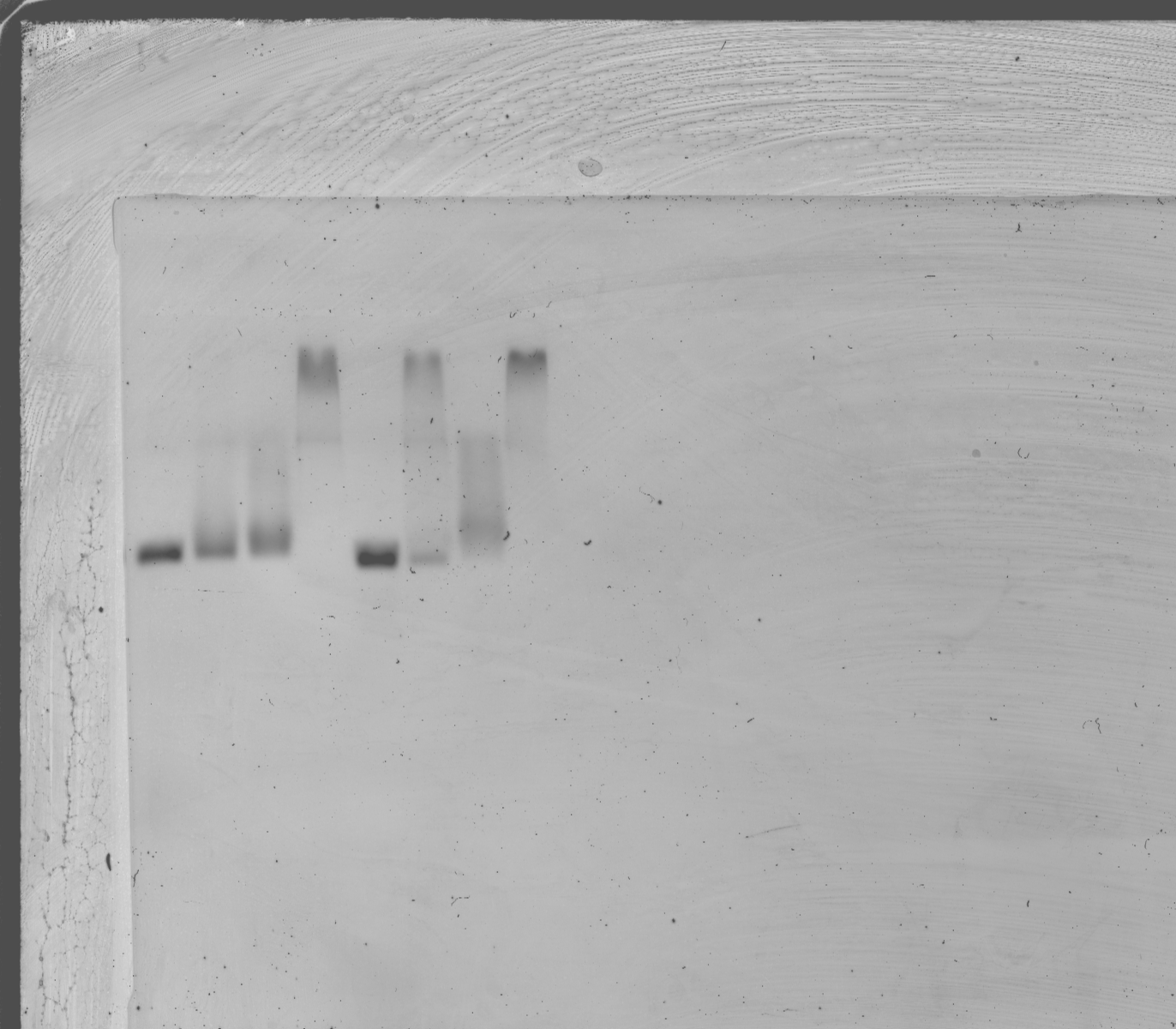


Figure 4B


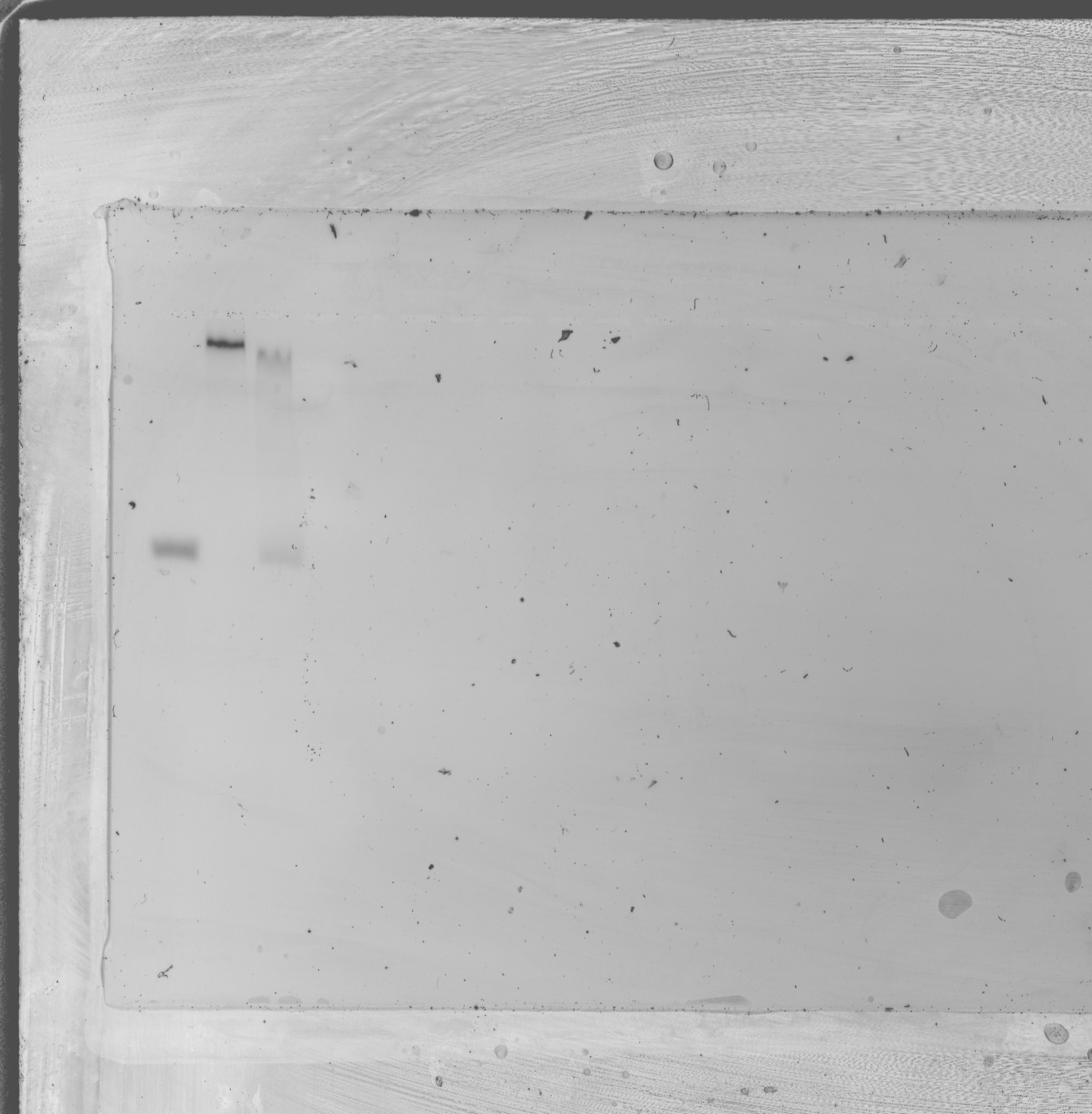

Supplement: Supplementary file 1 — Supplementary Materials [file 41598_2018_35844_MOESM1_ESM.docx]
